# A New Renieramycin T Right-Half Analog as a Small Molecule Degrader of STAT3

**DOI:** 10.3390/md22080370

**Published:** 2024-08-14

**Authors:** Preeyaphan Phookphan, Satapat Racha, Masashi Yokoya, Zin Zin Ei, Daiki Hotta, Hongbin Zou, Pithi Chanvorachote

**Affiliations:** 1 Center of Excellence in Cancer Cell and Molecular Biology, Faculty of Pharmaceutical Sciences, Chulalongkorn University, Bangkok 10330, Thailand; p.phookphan@gmail.com (P.P.); satapatto@gmail.com (S.R.); hushushin@gmail.com (Z.Z.E.); 2 Department of Pharmacology and Physiology, Faculty of Pharmaceutical Sciences, Chulalongkorn University, Bangkok 10330, Thailand; 3 Interdisciplinary Program in Pharmacology, Graduate School, Chulalongkorn University, Bangkok 10330, Thailand; 4 Department of Pharmaceutical Chemistry, Meiji Pharmaceutical University, 2-522-1, Noshio, Kiyose, Tokyo 204-8588, Japan; yokoya@my-pharm.ac.jp (M.Y.); 0eh3264t133443f@au.com (D.H.); 5 College of Pharmaceutical Sciences, Zhejiang University, Hangzhou 310058, China; zouhb@zju.edu.cn; 6 Faculty of Pharmacy, Silpakorn University, Nakhon Pathom 73000, Thailand

**Keywords:** renieramycin derivatives, structure–activity relationship, synthesis, NSCLC, STAT3, EMT, degradation, molecular docking

## Abstract

Constitutive activation of STAT3 contributes to tumor development and metastasis, making it a promising target for cancer therapy. (1R,4R,5S)-10-hydroxy-9-methoxy-8,11-dimethyl-3-(naphthalen-2-ylmethyl)-1,2,3,4,5,6-hexahydro-1,5-epiminobenzo[d]azocine-4-carbonitrile, DH_31, a new derivative of the marine natural product Renieramycin T, showed potent activity against H292 and H460 cells, with IC_50_ values of 5.54 ± 1.04 µM and 2.9 ± 0.58 µM, respectively. Structure–activity relationship (SAR) analysis suggests that adding a naphthalene ring with methyl linkers to ring C and a hydroxyl group to ring E enhances the cytotoxic effect of DH_31. At 1–2.5 µM, DH_31 significantly inhibited EMT phenotypes such as migration, and sensitized cells to anoikis. Consistent with the upregulation of ZO1 and the downregulation of Snail, Slug, N-cadherin, and Vimentin at both mRNA and protein levels, in silico prediction identified STAT3 as a target, validated by protein analysis showing that DH_31 significantly decreases STAT3 levels through ubiquitin-proteasomal degradation. Immunofluorescence and Western blot analysis confirmed that DH_31 significantly decreased STAT3 and EMT markers. Additionally, molecular docking suggests a covalent interaction between the cyano group of DH_31 and Cys-468 in the DNA-binding domain of STAT3 (binding affinity = −7.630 kcal/mol), leading to destabilization thereafter. In conclusion, DH_31, a novel RT derivative, demonstrates potential as a STAT3-targeting drug that significantly contribute to understanding of the development of new targeted therapy.

## 1. Introduction

Lung cancer is the leading cause of cancer-related mortality [1]. The survival rate for patients with non-small cell lung cancer (NSCLC), which accounts for nearly 80% of all lung cancer cases, is strongly influenced by the presence of metastasis [2].

Accumulated research over the past decade has highlighted the importance of an epithelial-to-mesenchymal transition (EMT) mediated by increasing master EMT transcription factors, namely Snail, Slug, and Twist, on the metastasis potential of human cancers [3,4,5]. These EMT factors, regulated by signal transducers and activators of transcription 3 (STAT3), play a crucial role in facilitating the rapid transition of cells between epithelial and mesenchymal phenotypes [6]. The STAT3 is found at high levels in patients with NSCLC, and is associated with advanced disease states in patients, such as increased metastasis and poorer prognosis [7].

Numerous molecules, including STAT3, are targeted by the ubiquitin–proteasome system [8], which is involved in the regulated breakdown of selective proteins. STAT3 degraders that promote the ubiquitination of STAT3 have been found to inhibit the growth of cancer cell lines and achieve tumor regression [9,10]. Thus, enhancing STAT3 ubiquitination in cancers represents a promising therapeutic strategy [9,11].

Recent studies on drug targets have revealed that both natural products and chemical compounds have the potential to inhibit the function of STAT3 by binding to the DNA binding domain or the Src Homology 2 (SH2) domain, promoting STAT3 ubiquitination and degradation [12,13,14]. However, various STAT3 inhibitors, including marine natural products, have been designed to target the SH2 domain, containing the Tyr705 residue that is important for STAT3 activation and dimerization [12,15,16,17]. The primary functions of DNA-binding domain are binding to the activation sites within the promotor regions of specific target genes, leading to transcription. Consequently, blocking the function of DNA-binding domain is one of the most important strategies for developing STAT3 direct inhibitors [18]. Therefore, the development of drugs to inhibit the DNA-binding domain of STAT3 may represent a better approach [14].

Interestingly, recent study found that reneiramycin T (RT) reduced STAT3 expression in B16F10 cells induced by the RAW264.7 supernatant [19]. However, the yield of RT is very limited. Previous studies reported that RT was prepared from renieramycin M (RM) by a photoredox reaction with a 64% overall yield [20], while RM (681 mg) was isolated from the marine sponge *Xestospongia* sp. (8.4 kg wet weight) [21]. Although RT and RM can be synthesized, the process is lengthy. The synthesis of RM had an overall yield of 3.9% over 17 steps in the longest linear sequence [22], while RT required 22 steps with a 6.2% overall yield [23].

To overcome these challenges, we synthesized right-half [24,25,26] and left-half [27] derivatives of RT and studied their anticancer activities and mechanisms of action to improve the structure–activity relationship. Among them, the right-half derivatives of RT showed higher amounts of anticancer activity than the left-half [26,27]. Not only did RM show activities against cancer stem cells, but the right-half derives of RT, DH_32, suppressed colony and spheroid formation by the downregulation of ALDH1A1, CD133, and CD44 via β-catenin [25]. Additionally, a previous study also showed that the right-half models of RT such as TM-(−)-18 increased apoptosis in a concentration-dependent manner and had binding affinity to MCL1 similar to RT [28]. However, there are no information on the effects of the simplified right-half model of RT compounds on STAT3 and EMT-mediated metastasis.

To investigate the effect of RT right-half analogs on STAT3 in lung cancer cells, we synthesized new right-half derivatives of RT, modifying the C- and E-ring parts based on SAR. In these analogs, the phenyl group in ring C was replaced with the pyridine, thiazole, or naphthalene rings, and the benzyl group in ring E was replaced with the hydroxyl group. We assessed the antimetastatic potential of a newly synthesized right-half RT derivative in NSCLC cells, including the inhibition of cell migration, invasion, and anoikis resistance. Additionally, we employed an in silico approach to predict target proteins and explored the underlying mechanisms, STAT3 and EMT markers, using immunofluorescence, Western blot analysis, Real-time qPCR, ubiquitin–proteasomal degradation assays, and molecular docking simulations. This study also has broader implications, ultimately supporting the pharmaceutical significance of synthetic compounds and SAR studies on target proteins.

## 2. Results

### 2.1. Synthesis of Seven Derivatives of RT Right-Half Analog

**Figure 1:**
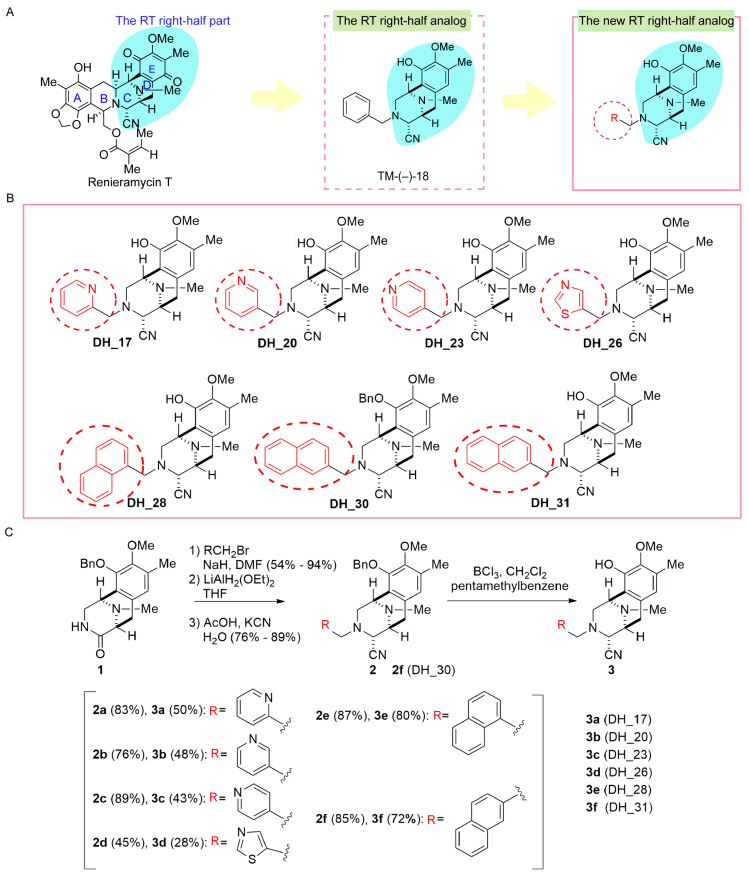
Derivatives of the RT right-half analogs—DH_17, DH_20, DH_23, DH_26, DH_28, DH_30, and DH_31. (**A**) The structure of Renieramycin T, TM-(−)-18, and the core structure of the RT right-half analog with R. R represents the position of the pyridyl, thiazolyl, or naphthalenyl group in ring C of the RT right-half analog, respectively. (**B**) Structures of the present RT right-half analogs: DH_17, DH_20, DH_23, DH_26, DH_28, DH_30, and DH_31. (**C**) Step-by-step synthesis for derivatives of RT right-half analogs.

According to our previous structure–activity relationship (SAR) information, an arylmethyl group linked to the nitrogen atom of the C-ring of the RT right-half analogs, such as benzyl, and the existence of aminonitrile in the fused CDE-ring system are essential for generating strong antitumor activity (Figure 1A) [26]. Additionally, converting benzyl ether to phenol, corresponding to the E ring of the RT right-half analog, enhances its anticancer activity [26]. Our recent studies demonstrated that analogs incorporating the pyridyl, thiazolyl, or naphthalyl group as the R in ring C of the RT right-half analog exhibited cytotoxicity against cancer cells [24,25]. Therefore, in this study, we focus on the right-half model of RT that converts the benzyl ether to phenol in the E ring and incorporates the pyridyl, thiazolyl, or naphthalyl group in the ring C of the RT right-half analog. The new derivatives of the RT right-half model, namely DH_17, DH_20, DH_23, DH_26, DH_28, DH_30, and DH_31, are shown in Figure 1B. These derivatives were synthesized for the anticancer activity assessments. The step-by-step process of the asymmetric synthesis of derivatives is shown in Figure 1C. The methods for synthesizing compounds **2a**–**2d** have been documented in the study conducted by Petsri et al. [24]. Consequently, the deprotected compounds **3a**–**3d** of previously synthesized compounds **2a**–**2d** with heterocyclic rings were synthesized. Additionally, **2e** and **2f** with the phenyl ring replaced by a naphthalene ring, along with their corresponding deprotected phenols **3e** and **3f**, were synthesized. Asymmetric synthesis of **2e** and **2f**, featuring a naphthalene ring, was performed in two steps (1: N-alkylation, 2: reductive cyanation of lactam carbonyl) in accordance with previous methods, starting from the common key intermediate 1, which can be easily synthesized from L-tyrosine. As shown in Figure 1C, compounds **2a**–**2f** were debenzylated using BCl_3_ in the presence of pentamethylbenzene, and the corresponding phenols, **3a**–**3f**, were synthesized [29].

### 2.2. Derivatives of RT Right-Half Analog Induce Cytotoxicity and Apoptosis

**Figure 2:**
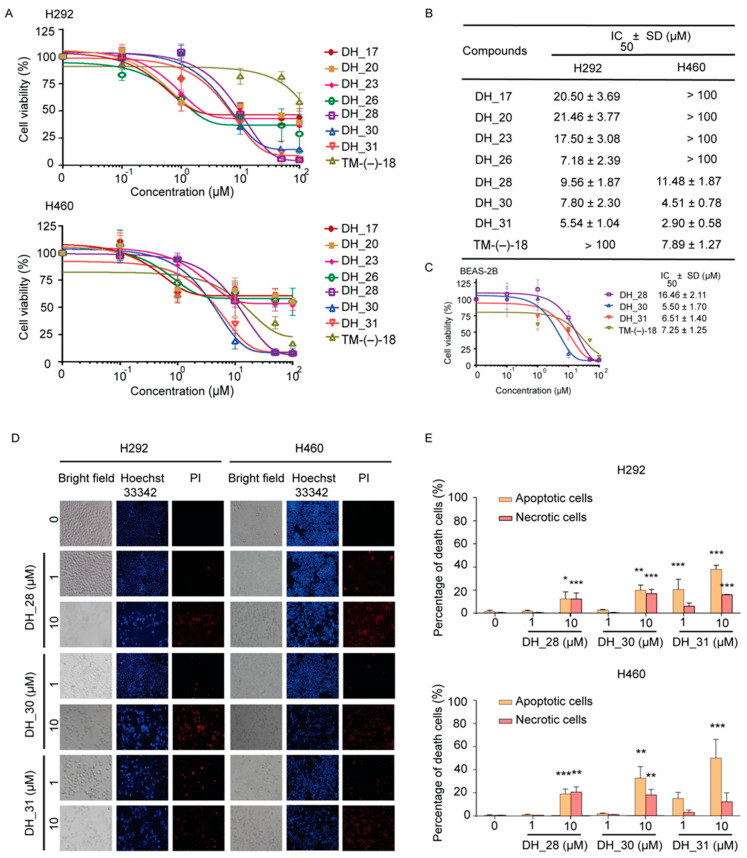
The effect of RT right-half analogs on cytotoxicity in NSCLC and human normal lung epithelial (BEAS-2B) cell lines and apoptotic cell death in NSCLC cells. (**A**) NSCLC H292 and H460 cells were treated with derivatives of RT right-half analogs for 24 h and analyzed using MTT assay to assess cytotoxicity. (**B**) IC_50_ values for H292 and H460 cell lines were calculated. (**C**) BEAS-2B cells were treated with DH_28, DH_30, DH_31, and TM-(−)-18 for 24 h. The cytotoxic effects were evaluated using an MTT assay, and the IC_50_ values for BEAS-2B cells were determined. (**D**) H292 and H460 cells were seeded and treated with 0–10 μM of DH_28, DH_30, and DH_31 for 24 h. Hoechst 33342 and PI were used to stain the cell nuclei. Images were obtained under a fluorescence microscope. (**E**) The percentages of cell death were calculated based on the stained images in H292 and H460 cells. Data represent the mean ± SD (*n* = 3). *, **, and *** indicate a statistically significant difference between the treated and the untreated cells at *p* < 0.05, *p* < 0.01, and *p* < 0.001, respectively.

To determine the most effective small molecules for further experiments, the cytotoxicity of each compound was assessed using an MTT assay. NSCLCs H292 and H460 were treated with various concentrations of derivatives of the RT right-half analog for 24 h. The cytotoxicity profiles are presented in Figure 2A,B. The results show that the compounds DH_17, DH_20, DH_23, and DH_26 reduced the viability of H292 cells, with half-maximal inhibitory (IC_50_) values of 20.50 ± 3.69 µM, 21.46 ± 3.77 μM, 17.50 ± 3.08 μM, and 7.18 ± 2.39 μM, respectively. However, the IC_50_ values for DH_17, DH_20, DH_23, and DH_26 in H460 cells were greater than 100 μM. TM-(−)-18 was identified as a potent compound in a previous study [28]. The structure of TM-(−)-18 was present in Figure 1A. When compared to TM-(−)-18, DH_28, DH_30, and DH_31 exhibited a more potent cytotoxic effect in both H292 and H460 cells, with IC_50_ values lower than 15 µM (Figure 2B). Among these three compounds, DH_31 was the most effective, with IC_50_ values of 5.54 ± 1.04 µM and 2.9 ± 0.58 µM in NSCLCs H292 and H460, respectively (Figure 2B). To assess the selectivity of DH_28, DH_30, DH_31, and TM-(−)-18 in lung cancer cells, non-tumorigenic epithelial cell lines derived from human bronchial epithelial cells (BEAS-2B) were treated with DH_28, DH_30, DH_31, and TM-(−)-18 at various concentrations for 24 h. The IC_50_ values for DH_28, DH_30, DH_31, and TM-(−)-18 were 16.46 ± 2.11 µM, 5.50 ± 1.70 μM, 6.51 ± 1.40 μM, and 7.25 ± 1.25 μM, respectively (Figure 2C). Interestingly, the IC_50_ values of DH_28 and DH_31 in BEAS-2B cells were higher than those of NSCLC cells, whereas the IC_50_ value of TM-(−)-18 in BEAS-2B cells was lower and similar to that in H292 and H460 cells, respectively.

Apoptosis is a crucial mechanism of cell death induced by anticancer drugs. We further analyzed the apoptosis induction effect of the potent small molecules, namely DH_28, DH_30, and DH_31. Nuclear morphology detected by the staining with nucleus-specific fluorescence dye Hoechst 33342 and cell-impermeable PI for necrotic detection was performed. Morphologic changes by means of condensed and/or fragmented nuclei were observed after 24 h of treatment (Figure 2D). As shown in Figure 2E, the results showed that statistically significant cytotoxic effects of DH_28, DH_30, and DH_30 were observed at a concentration of 10 μM in both H292 and H460 cells. In H292 cells, the apoptosis results showed significant apoptosis at 10 µM for DH_28 (*p* < 0.05) and for DH_30 (*p* < 0.01), while DH_31 induced significant apoptosis at both 1 µM and 10 µM (*p* < 0.001). Necrosis was detected in H292 cells at 10 µM of DH_28, DH_30, and DH_31 (*p* < 0.001). H460 cell lines exhibited significant apoptotic cell death after treatment with 10 µM of DH_28 (*p* < 0.001), DH_30 (*p* < 0.01), and DH_31 (*p* < 0.001). Additionally, significant necrosis was observed in H460 cells treated with 10 µM of DH_28 (*p* < 0.01) and DH_30 (*p* < 0.05), while treatment with DH_31 at 1 µM and 10 µM did not cause significant necrosis. According to the cytotoxic and apoptosis results, DH_31, which contains a naphthalene ring, had a potent cytotoxic effect and caused 34–66% of the dead cells in NSCLC at 10 μM, which was higher than that of the other small molecules. This suggests that DH_31 could be a potent inhibitor of cancer cell survival. Therefore, DH_31 was selected for further experiments.

### 2.3. DH_31 Suppresses Metastasis Mechanism

**Figure 3:**
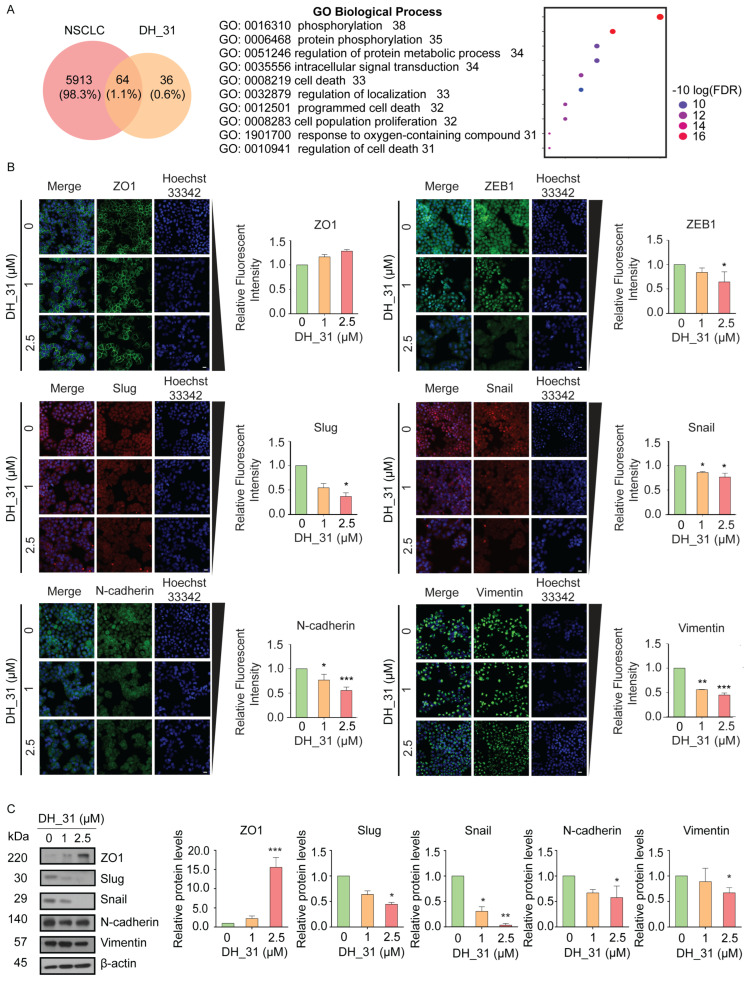
Putative analysis of NSCLC against DH_31 and the effect of DH_31 on EMT-association proteins. (**A**) Venn diagram of NSCLC and DH_31 targets and GO enrichment analysis of putative targets was performed to clarify the relevant biologic processes (*p* < 0.01). The y-axis represents GO terms, and the x-axis indicates the number of genes enriched in that term. The color from blue to red indicates the value of *p*. The adjust (FDR) is becoming smaller with greater credibility and importance. (**B**) The expression levels of ZO1, ZEB1, Slug, Snail, N-cadherin, and Vimentin were visualized by fluorescence microscopy. Scale bar, 20 µm. Bar graphs show the relative levels of ZO1, ZEB1, Slug, Snail, N-cadherin, and Vimentin. (**C**) The protein expression levels of ZO1, Slug, Snail, N-cadherin, Vimentin and β–actin were evaluated by Western blot analysis. The relative protein levels were calculated by densitometry. Data represent the mean ± SD (*n* = 3). *, **, and *** indicate a statistically significant difference between the treated and untreated cells at *p* < 0.05, *p* < 0.01, and *p* < 0.001, respectively.

To investigate the possible mechanism of DH_31 on NSCLC, computational methods for NSCLC-associated genes or proteins and drug target prediction was performed [30]. NSCLC-associated genes were used as described in a previous study [31]. The target list of DH_31 was retrieved from the Swiss Target Prediction database, which provides ligand-based target prediction [32]. Venn diagram analysis showed that the NSCLC-associated genes were associated with 5977 targets, while DH_31 interacted with 100 targets. The intersection analysis revealed 64 overlapping targets between DH_31 and the NSCLC-associated genes (Figure 3A). The list of 64 targets is provided in the Supporting Information, Table S2. The list of 64 core genes.

To understand the biological functions of 64 highlighted targets of DH_31, enrichment analysis was performed using ShinyGO 0.80 that contains functional annotation Gene Ontology (GO) terms [33]. The top 10 terms were chosen based on their number of genes in that term (*p* < 0.01). Detailed information of GO analyses is shown in the Supporting Information, Table S3. The top 10 significantly enriched GO term. The results indicated that biological processes of DH_31 on NSCLC were involved in the phosphorylation (GO: 0016310), protein phosphorylation (GO: 0006468), regulation of protein metabolic process (GO: 0051246), intracellular signal transduction (GO: 0035556), cell death (GO: 0008219), the regulation of localization (GO: 0032879), programmed cell death (GO: 0012501), cellular population proliferation (GO: 0008283), the response to the oxygen-containing compound (GO: 1901700), and the regulation of cell death (GO: 0010941). Our enrichment analysis also showed that half of target proteins of DH_31 are involved in the regulation of localization, programmed cell death, and protein metabolic process.

EMT is a biological process that is associated with cancer progression and the spread of cancer cells to other parts of the body. This mechanism facilitates the motility of cancer cells [34], enhances anoikis resistance [35], and contributes to poor prognosis due to its high metastatic potential. Given the critical role of EMT in the metastasis processes, we firstly investigated EMT markers. Therefore, to validate that DH_31 inhibits metastasis, H460 cells were selected to elucidate the mechanism because of their enhanced migration and invasion behavior [36]. H460 cells were exposed to the non-toxic concentration range of 0–2.5 µM of DH_31 for 24 h. Subsequently, the evaluation of EMT expression levels was carried out through the application of immunofluorescence assay and Western blotting. We further investigated key hallmarks of EMT, specifically the reduction in the expression levels of epithelial marker (ZO1) and the increase in the expression levels of mesenchymal markers (Slug, Snail, ZEB1, N-cadherin, and Vimentin) [37]. The results of immunofluorescence staining indicated that DH_31 slightly increased the levels of ZO1 and decreased the levels of mesenchymal markers (Figure 3B). DH_31 treatment led to a significant decrease in ZEB1 and Slug protein levels at 2.5 μM (*p* < 0.05). The expressions of Snail, N-cadherin, and Vimentin were reduced in a concentration-dependent manner. Notably, a significant reduction was observed in levels of Snail at 1 μM and 2.5 μM (*p* < 0.05), N-cadherin at 1 μM (*p* < 0.05) and 2.5 μM (*p* < 0.001), and Vimentin at 1 μM (*p* < 0.01) and 2.5 μM (*p* < 0.001). Furthermore, the results from the Western blot analysis confirmed the findings from immunofluorescence staining (Figure 3C). The treated cells showed a significant decrease in ZO1 expression at 2.5 μM (*p* < 0.001). Additionally, reductions in the expression of Slug, N-cadherin, and Vimentin were observed at 2.5 µM (*p* < 0.05), while the expression of Snail was reduced at 1 µM (*p* < 0.05), and 2.5 µM (*p* < 0.01). The protein alterations in response to DH_31 were clarified. DH_31 downregulated the expression of the mesenchymal markers of Slug, Snail, N-cadherin, and Vimentin, but increased the expression of the epithelial marker ZO1.

### 2.4. DH_31 Inhibits Migration and Increases Sensitivity to Anoikis

Since we demonstrated that DH_31 inhibits EMT markers, we next determined the effect of DH_31 on cell migration. The metastatic cascade is a multistage progression wherein cancer cells detach from the primary tumor, migrate, and invade the surrounding microenvironment to colonize secondary organs [38]. To explore whether DH_31 could suppress these aggressive behaviors, wound healing assays and anoikis assays were evaluated, respectively.

**Figure 4:**
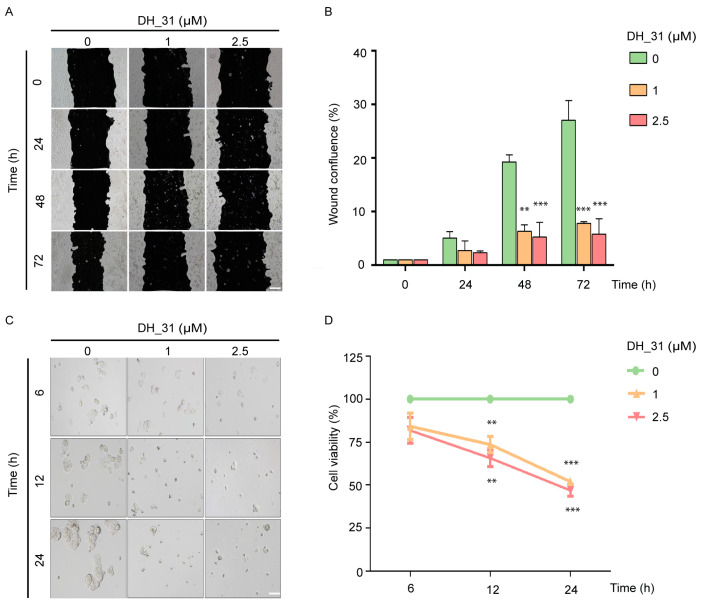
The effects of DH_31 on migration and anoikis resistance on NSCLC H460. (**A**) DH_31 decreased the migration of H460 cells. (**B**) The relative migration levels of the treated and untreated cells were determined at 24, 48, and 72 h. (**C**) DH_31 increased the sensitivity to anoikis in H460 cells. (**D**) The relative viability of cells was determined after culture under detachment conditions for 6, 12, and 24 h. Scale bar, 20 µm. Data represent the mean ± SD (*n* = 3). ** and *** indicate a statistically significant difference between the treated and the untreated cells at *p* < 0.01 and *p* < 0.001, respectively.

As shown in Figure 4A, the wound healing assay demonstrated that DH_31 attenuated the migratory activity of H460 cells in a concentration-dependent manner during the experimental period. Figure 4B shows that DH_31 significantly decreased cell migration across the wound space at 48 h (*p* < 0.01 for 1 μM and *p* < 0.001 for 2.5 μM), and 72 h (*p* < 0.001 for both 1 and 2.5 μM), compared to untreated cells.

Anoikis, a detachment-induced apoptosis, is a mechanism to prevent metastasis, so resistance to anoikis enhances the potential of metastasis [34]. To confirm that DH_31 induces anoikis, the detached H460 cells were treated with 1 and 2.5 μM of DH_31 for 6, 12, and 24 h. Untreated H460 cells spontaneously formed multicellular aggregations after 6 h, while cells mostly exhibited a single cell pattern after treatment with DH_31 (Figure 4C). A significant decrease in the cell viability of detached H460 cells by DH_31 was observed in a concentration- and time-dependent manner at 12 h (*p* < 0.01) and 24 h (*p* < 0.001), as compared with untreated cells (Figure 4D).

### 2.5. DH_31 Decreases STAT3 Protein Expression

**Figure 5:**
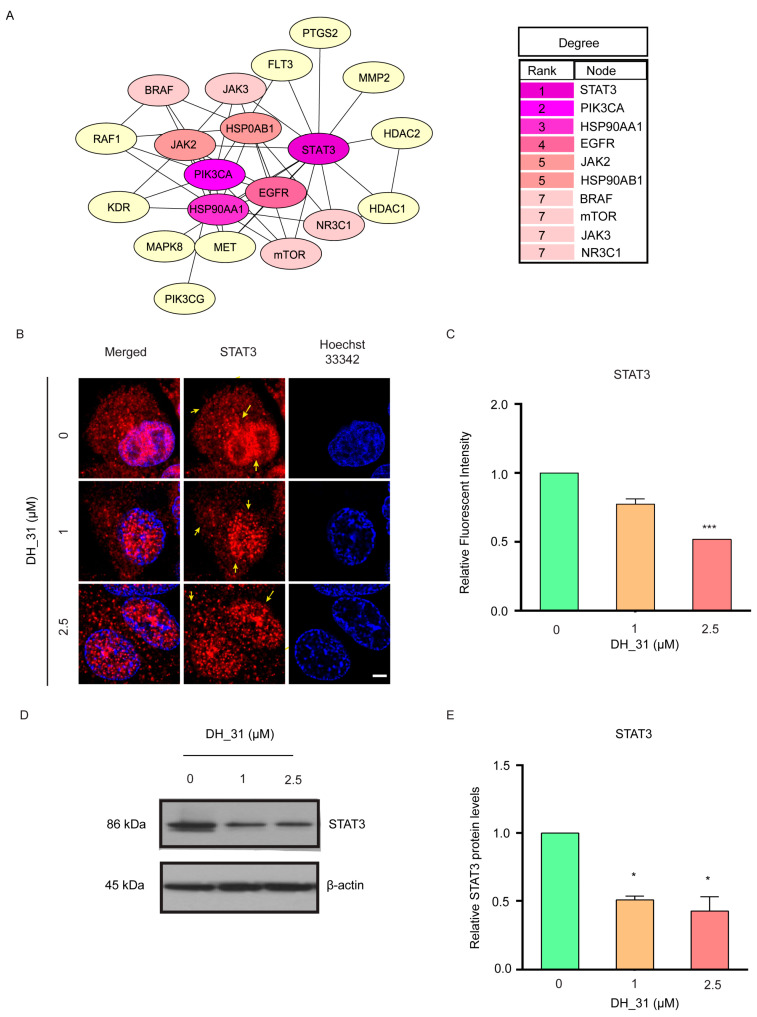
STAT3 identified as a potential target of DH_31. (**A**) The top 10 targets among the 64 targets were ranked based on the number of degrees, visualized by the CytoHubba plugin. The degree values of the top 10 targets in the PPI network were ranked, with STAT3 having the highest degree. The intensity of the colors corresponded to the degree values, with purple indicating large values, pink indicating moderate values, and yellow indicating small values. (**B**) H460 cells treated with DH_31 (0–2.5 μM) for 24 h were stained with anti-STAT3 antibody (red) and examined using confocal laser scanning microscopy. Cell nuclei were stained with Hoechst 33342 (blue). Scale bar, 10 µm. Arrows denote localized STAT3 proteins. (**C**) The relative levels of STAT3 of H460 were determined by immunofluorescence analysis. (**D**) The protein expression levels of STAT3 and β–actin was evaluated by Western blot analysis. (**E**) The relative protein levels were calculated by densitometry. Data represent the mean ± SD (*n* = 3). * and *** indicate a statistically significant difference between the treated and untreated cells at *p* < 0.05 and *p* < 0.001, respectively.

Following the validation of the ability of DH_31 to suppress EMT markers, cell mobility, and anoikis resistance (Figure 3B,C and Figure 4), 64 predicted targets (Figure 3A) were identified through the PPI (Protein–Protein Interaction) network. A PPI network in STRING is a comprehensive database and web resource that visualizes known and predicted interactions between proteins [39]. As shown in Figure 5A, these target genes were ranked according to their degree count, with the top 10 targets in the PPI network being identified. Among these targets, STAT3 exhibited the highest score (Figure 5A), suggesting it as the primary target for further experimentation.

To explore whether DH_31 could suppress STAT3, H460 cells were treated with 1 and 2.5 μM of DH_31 for 24 h. Immunofluorescence and confocal microscopy were performed to visualize STAT3 expression. As shown in Figure 5B, in untreated H460 cells, STAT3 was predominantly localized in the nucleus, consistent with its role as a transcription factor. Following DH_31 treatments, reductions were observed in both cytoplasmic and nuclear STAT3 levels. Statistical analysis confirmed a significant reduction in STAT3 expression at 2.5 µM of DH_31 (*p* < 0.001) (Figure 5C).

The results from the Western blot analysis also showed that DH_31 reduced the expression of STAT3 proteins (Figure 5D,E), with a significant decrease in STAT3 levels at 1 μM and 2.5 μM (*p* < 0.05). These findings imply that DH_31 exerts its anticancer effects by modulating the expression and/or accumulation of STAT3.

### 2.6. DH_31 Decreases STAT3 through the Induction of STAT3 Proteasomal Degradation

**Figure 6:**
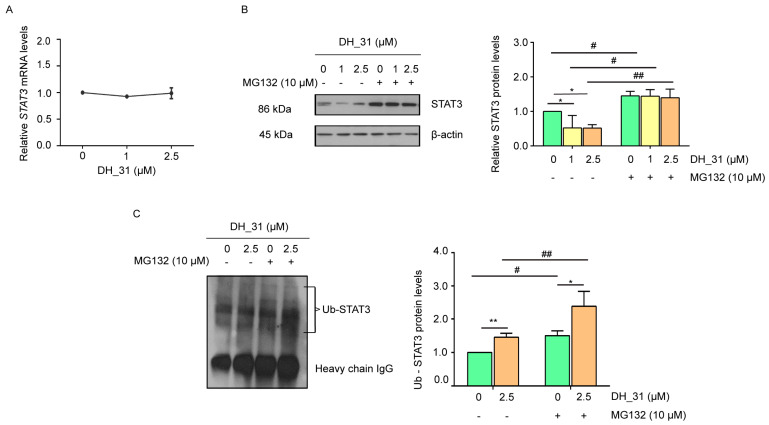
The effect of DH_31 on enhanced ubiquitin-mediated STAT3 proteasomal degradation in NSCLC H460. H460 cells were treated with DH_31 (0–2.5 μM) for 8 h. (**A**) The expression levels of STAT3 mRNA were determined by Real-time qPCR. (**B**) The ubiquitin–proteasome inhibitor MG132 reversed the inhibitory effect of DH_31 on the expression of the STAT3 protein. After treatment with or without MG132 (10 µM) for 1 h, cells were treated with DH_31 (0–2.5 µM) for 6 h. The STAT3 levels were measured using Western blot analysis and calculated by densitometry. (**C**) DH_31 induced the ubiquitin–proteasomal degradation of STAT3. After treatment with or without MG132 (10 µM) for 1 h, cells were treated with DH_31 (0 and 2.5 µM) for 6 h. The protein lysates were collected subsequent to STAT3 immunoprecipitation, and the ubiquitinated protein levels were measure by Western blot analysis. Ub-STAT3 levels were calculated by densitometry. Data represent the mean ± SD (*n* = 3). *, and ** indicate a statistically significant difference between the treated and untreated cells at *p* < 0.05 and *p* < 0.01, respectively. # and ## indicate a statistically significant difference from the cells without MG132 at *p* < 0.05 and *p* < 0.01, respectively.

To explore whether the reduction in total STAT3 protein after DH_31 treatments (as shown in Figure 5B–E) occurs at the mRNA or protein levels, we first investigated mRNA levels using Real-time qPCR. The result showed that the mRNA expression of STAT3 did not change significantly after treatment with DH_31 for 8 h (Figure 6A). Although DH_31 treatments did not affect the mRNA level of STAT3, the protein expression of EMT markers exhibited significant alterations (Figure 3B,C). This finding suggests that these effects could be attributed to the level of STAT3 protein. To evaluate whether DH_31 affects STAT3 stability, pretreatment with MG132, a potent selective proteasome inhibitor, was performed. H460 cells were pretreated with or without MG132 (10 μM) for 1 h and then cells were either treated with DH_31 for 6 h or left untreated. As shown in Figure 6B, DH_31 significantly reduced the STAT3 protein levels at 1 and 2.5 µM for the treated cells compared to the untreated cells (*p* < 0.05), while pretreatment with MG132 significantly reversed DH_31-induced STAT3 downregulation compared to the cells not pretreated with MG132 at 0 (*p* < 0.05), 1 (*p* < 0.05) and 2.5 µM (*p* < 0.01). Treatment with MG132 at all concentrations drastically increased the STAT3 level and no significant difference was observed between these groups, which confirmed that the STAT3 protein was degraded mainly through the proteasomal degradation pathway. The proteasome can recognize and degrade protein targets that are conjugated by single or poly-ubiquitin molecules. To investigate the ubiquitination level of STAT3 with DH_31 treatments, the ubiquitination level was evaluated using immunoprecipitation and Western blot analysis of the STAT3–ubiquitin complex in the lung cancer cells treated with DH_31 and in untreated cells. Additionally, Figure 6C shows that the cells with the DH_31 treatment significantly enhanced STAT3–ubiquitin formation compared to untreated cells with MG132 (*p* < 0.05) and untreated cells that were not pretreated with MG132 (*p <* 0.01). These results indicated that DH_31 promotes STAT3 degradation via the ubiquitin–proteasomal pathway without affecting STAT3 transcription in H460 cells.

### 2.7. DH_31 Interactions with STAT3 Protein

**Figure 7:**
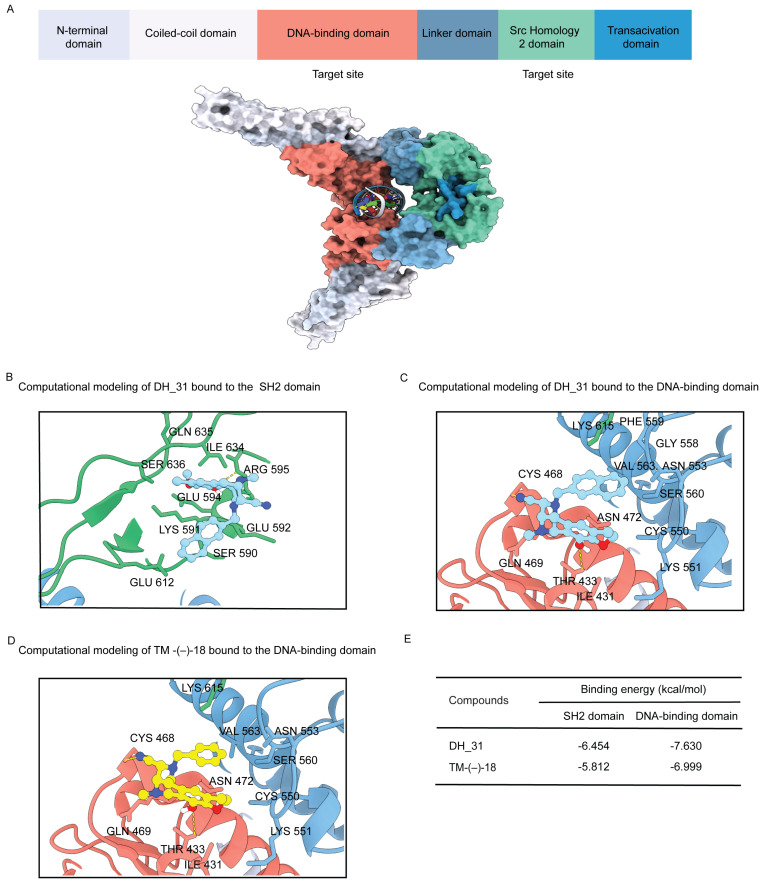
Domain structure of STAT3 and structure of DH_31 with in silico predicted binding configurations. (**A**) Schematic of the domain structure of STAT3 and the structure of the dimer interface of STAT3 (PDB: 1BG1) illustrating the surface locations of the DNA-binding domain (residues 321–494) (red) and the SH2 domain (residues 584–688) (green), (**B**) the binding interaction of DH_31 to the SH2 domain of STAT3, (**C**) the binding interaction of DH_31 to the DNA-binding domain, and (**D**) the binding interaction of TM-(−)-18 to the DNA-binding domain of STAT3. (**E**) The binding energy of DH_31 and TM-(−)-18 at the SH2 domain and the DNA-binding domain.

To provide a better understanding of the mechanism by which DH_31 modulates STAT3 stability, molecular docking was performed to investigate possible interactions. The most common approach in targeting STAT3 directly is to prevent the formation of functional STAT3 dimers through disrupting the domains of SH2 [12,15,16]. Additionally, targeting STAT3 through its DNA-binding domain interactions represents a novel approach for pharmacological intervention against cancers driven by constitutive STAT3 activation [14,15,40,41]. Therefore, we compared the binding of DH_31 with the SH2 domain with that for the DNA-binding domain of STAT3 to identify the most effective binding site. STAT3 is characterized by the presence of six different functional domains (Figure 7A). The SH2 domain comprises three sub-pockets [15], and the binding modes of DH_31 reveal its occupancy in all three. DH_31 exhibited a binding affinity of −6.454 kcal/mol. It interacted with residues Lys591 and Glu612 in the phosphorylated Tyr705 binding pocket, along with residues Ile634, Gln635, and Ser636 in the Leu706 subsite. Additionally, it interacted with residues Ser590, Glu592, and Glu594, forming a hydrogen bond with Arg595 in a hydrophobic side pocket (Figure 7B).

For the DNA-binding domain, DH_31 exhibited a binding affinity of −7.630 kcal/mol, which was stronger than that of the SH2 domain (−6.454 kcal/mol). DH_31 formed a hydrogen bond with residue Thr433 and interacted with residues Ile431, Gln469, Asn472, Cys550, Lys551, Asn553, Gly558, Phe559, Ser560, Val563, and Lys615. Additionally, molecular docking studies indicated that DH_31 had the potential to covalently bind to STAT3, as the cyano group of DH_31 closely approached the thiol group of Cys468 in the DNA-binding domain of STAT3 (Figure 7C). The results of comparative docking studies between the SH2 domain and the DNA-binding domain suggest that DH_31 exhibits a stronger binding affinity for the DNA-binding domain of STAT3 compared to its SH2 domain.

To investigate the effect of the naphthalene ring of DH_31 in promoting the interaction between the cyano group of the RT right-half analog and the thiol group of Cys468 in the DNA-binding domain of STAT3, TM-(−)-18 was investigated (Figure 7D). The docking results showed that TM-(−)-18 had a binding affinity of −6.999 kcal/mol for the DNA-binding domain, which was weaker than that of DH_31 (−7.630 kcal/mol), (Figure 7E). This result revealed that the naphthalene ring of DH_31 is crucial for binding to STAT3, stabilizing the interaction more effectively than TM-(−)-18, and facilitating the covalent binding of the cyano group to Cys468 within the DNA-binding domain.

Our study suggests that the naphthalene ring of DH_31 is crucial for binding to STAT3, stabilizing the interaction more effectively than TM-(−)-18.

### 2.8. DH_31 Suppresses mRNA Expression of EMT Markers

**Figure 8:**
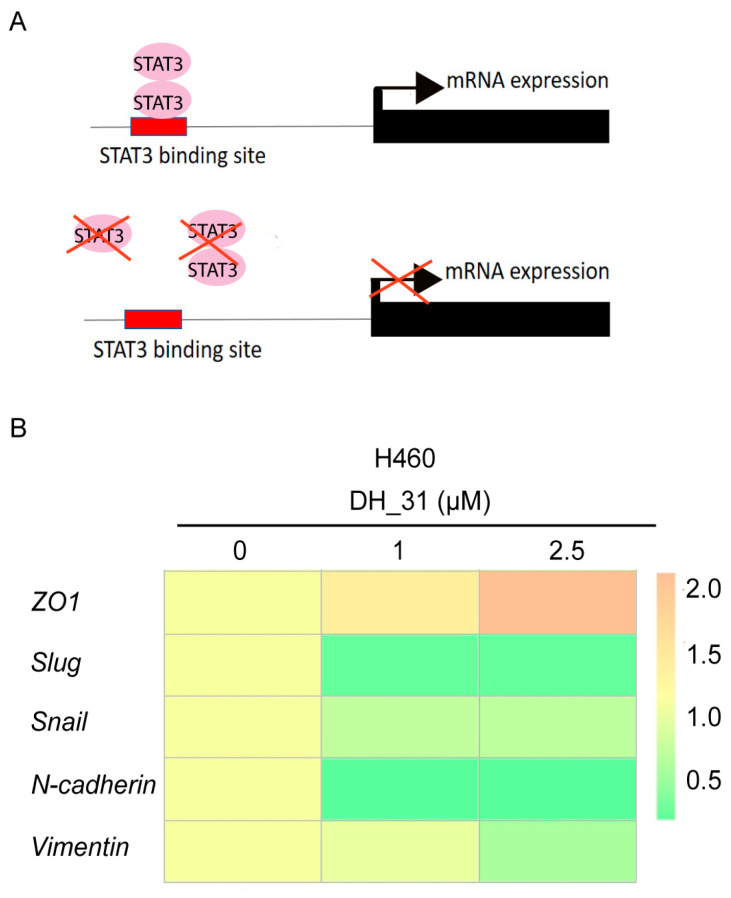
The effect of DH_31 on the mRNA expression of EMT markers in NSCLC H460. (**A**) Schematic representation of the of STAT3 transcription factor binding sites in target genes. (**B**) The mRNA expression of *ZO1*, *Slug*, *Snail*, *N-cadherin*, and *Vimentin* in H460 cells treated with DH_31 (0–2.5 µM).

Molecular docking analysis indicated that DH_31 showed a strong binding affinity to the DNA-binding domain of STAT3. Therefore, the effect of DH_31 on targeting, the DNA-binding domain of STAT3, may represent an impact on downstream targets at the transcriptional level (Figure 8A). The expression levels of *ZO1*, *Slug*, *Snail*, *N-cadherin*, and *Vimentin* were analyzed using Real-time qPCR. The heatmap illustration in Figure 8B indicates that DH_31 treatments at 1 μM and 2.5 µM caused a significant reduction in *Slug*, *Snail*, *N-cadherin*, and *Vimentin* (*p* < 0.05) and caused a significant increase in *ZO1* (*p* < 0.01). Our results revealed that DH_31 did not affect the mRNA levels of *STAT3* (Figure 6A) but had an effect on downstream target genes.

## 3. Discussion

Cancer metastasis is considered to be a significant contributor to the high mortality rates associated with NSCLC [42]. Therefore, targeted therapies, which focus on the inhibition of the metastatic process, are essential for improving the clinical outcome [43]. In this study, we demonstrated that DH_31 inhibits migration, anoikis resistance, and downregulates the protein expression of STAT3 and its downstream targets involved in metastasis in non-small cell lung cancer. Specifically, we observed that DH_31 decreased the levels of Snail, Slug, N-cadherin, and Vimentin, while increasing ZO1 expression at both mRNA and protein levels. Moreover, we established that DH_31 facilitates the depletion of STAT3 by enhancing its ubiquitin–proteasomal degradation. Molecular docking showed that DH_31 stably binds to Cys468 within the DNA-binding domain of STAT3.

Our prior knowledge of the SAR has shown that the presence of aminonitrile in the fused CDE-ring system, which constitutes the right half of renieramycin, is necessary for a potent anticancer effect [26]. Additionally, Matsubara et al. found that replacing benzyl ether with phenol in the E ring of the RT right-half analog has been shown to increase its anticancer activity [26]. Petsri et al. also confirmed that TM-(−)-18, a right half analog of RT with phenol in the E ring, exhibits anticancer activity in both patient-derived primary lung cancer cells and H460 cells [28]. Recent studies have further developed C-ring analogs of the right half of RT and shown that some of them enhance cytotoxic effects on NSCLC cells and can target at specific pathways [24,25]. In these analogs, the phenyl group in the ring C was replaced with pyridine, thiazole, or naphthalene rings [24,25]. Therefore, we synthesized novel right-half derivatives of renieramycin by modifying both the C- and E-ring components, guided by SAR. Our cytotoxicity results indicated that DH_28, DH_30, and DH_31 exhibited a more potent cytotoxic effect on H292 and H460 cells (Figure 2A,B), with the phenyl group in ring C of DH_28, DH_30, and DH_31 replaced by naphthalen-2-ylmethyl. This finding was in agreement with previous studies indicating that naphthalen-2-ylmethyl substitution is essential for the cytotoxic potency [25]. Wright et al. also showed that lipophilic imidazolium salts containing naphthalene substituents bound in the one or two position of the naphthalene ring with either methyl or ethyl linkers demonstrated promising anticancer activity against all three lines tested (HCC817, H1975, and H460 cells) [44]. However, among these three compounds, DH_31 was the most potent, with IC_50_ values at 24 h of 5.54 ± 1.04 µM and 2.9 ± 0.58 µM on NSCLC cells H292 and H460, respectively (Figure 2B). In addition, when comparing the structure of DH_30 and DH_31 in the current study, the presence of hydroxyl in DH_31 improved the cytotoxic potency for H292 and H460 cells. This improvement may presumably be due to enhanced solubility. Our study suggests that these modifications in derivatives of RT right-half analogs may enhance drug solubility in lipophilic environments and mitigate aggregation in aqueous environments, leading to increased cytotoxic potential in lung cancer cells.

Moreover, several studies from our research group showed that the RT right-half analogs exhibited effective anticancer activity, such as inducing apoptosis by targeting MCL1 or p53, as well as inhibiting CSC activity by targeting the Akt signaling pathway or β-catenin. However, there are no reports on RT right-half analogs impact on the metastasis mechanism. Therefore, this study aimed to uncover whether synthesis derivatives of right-half of RT could regain sensitivity to anoikis, decrease metastasis, and shed light on the underlying mechanisms. To explore the possible mechanism of DH_31 on NSCLC, bioinformatics and network pharmacology were employed to leverage protein–small molecule interactions [32], network-based methods [45], and disease–protein interactions [46]. Gene Ontology (GO) functional enrichment analysis results showed that the biological processes affected by DH_31 on NSCLC included protein phosphorylation, the regulation of protein metabolic processes, and intracellular signal transduction. Additionally, these processes involved cell death, the regulation of localization, programmed cell death, cellular population proliferation, the response to oxygen-containing compounds, and the regulation of cell death. Wang et al. reported that genes related metastatic breast cancer are significantly enriched in the GO terms, including the negative regulation of the apoptotic process, the positive regulation of gene expression, the regulation of the apoptotic process, the negative regulation of programmed cell death, and the regulation of the protein metabolic process [47]. A meta-analysis of gene expression profiles from cancer metastases identified common features and pathways involved in metastasis, highlighting the enrichment of pathways related to cell motility and adhesion, including cell localization. Our enrichment analysis also showed that half of target proteins of DH_31 are involved in the regulation of localization, programmed cell death, and the protein metabolic process, implying that this drug may have activity in suppressing migration.

The EMT mechanism is known to be responsible for the motility of cancer cells [34], enhancing anoikis resistance [35], and cancer metastasis [48]. Previous studies have highlighted renieramycin’s anti-metastatic potential [19,49]. For instance, Yu et al. found that RT treatment reduced Twist, Snail, Vimentin, and N-cadherin expression, as well as the migration ability of B16F10 cells induced by the RAW264.7 supernatant [19]. Additionally, the 22-*O*-(*N*-Boc-L-glycine) ester of RM inhibited H460 cell motility, and altered EMT marker expressions [49]. The findings from this research imply that 2-Boc-Gly-RM hinders the migration of cells by suppressing the expression of EMT [49]. Our findings align with these studies; DH_31 significantly decreased cell migration and increased sensitivity to anoikis compared to untreated cells. DH_31 also reduced mesenchymal markers (Slug, Snail, N-cadherin, and Vimentin) and increased the epithelial marker ZO1. These results suggest that RT right-half analogs like DH_31 can inhibit cell metastasis initiation and anoikis resistance by downregulating EMT.

During EMT progression, EMT-associated transcription factors of the zinc finger protein SNAI1 (Snail), zinc finger protein SNAI2 (Slug), zinc finger E-box-binding homeobox 1 (ZEB1), and Twist families are upregulated by STAT3 signaling [50]. STAT3-binding sites have been identified: the *Slug* promoter, *Snail* promoter, and the antisilencer element of *Vimentin*. These sites have been identified in multiple cancer cells [51,52,53]. Metastasis cancer studies, including those of lung cancer, have demonstrated that STAT3 is essential for maintaining *Slug* [51,54], *Snail* [52,55], and *Vimentin* [56] expression. In lung cancer cells A549 and H460, microarray analysis revealed that Slug is a downstream effector molecule of CXCR4/STAT3 signaling [54]. Yan et al. also demonstrated that the knockdown of *DHX9*, a member of the DEAH-box family of RNA-dependent ATPases, leads to the activation of STAT3 and subsequently induces mRNA and protein levels of Snail and Vimentin in lung cancer cells A549 [56]. Moreover, in hepatocellular carcinoma cells, overexpression of STAT3 significantly reduced the expression of cell adhesion molecules but enhanced the expression of *N-cadherin* and *Vimentin* [57]. Thus, STAT3 plays a crucial role in facilitating the rapid transition of cells between epithelial and mesenchymal phenotypes.

Not only is STAT3 associated with metastasis [58,59], but its activation of STAT3 is also related to drug resistance in patients [60]. Over 50% of patients with NSCLC show high expression of STAT3 [58]. STAT3 is a latent transcription factor found in the cytoplasm of cells. It is activated by tyrosine (Y705) phosphorylation, leading to nuclear translocation, and acts as a transcription factor to induce the expression of the target gene [61]. However, unphosphorylated STAT3 monomers or dimers have been reported that can regulate gene expression [62,63]**,** and other position phosphorylation-independent functions have been unclear for STAT3 [64]. Therefore, this study investigated the effects of DH_31 on total STAT3 expression in H460 cells. Our findings are consistent with a previous study demonstrating that targeting STAT3 leads to the downregulation of the expression of the STAT3 protein, promotes the degradation of STAT3 ubiquitination, and inhibits cell metastasis in human glioblastoma cells [13]. STAT3 is recognized by a specific E3 ubiquitin ligase [65] and conjugated to polyubiquitin, which is then degraded by the proteasome [66]. These changes in the fates of STAT3 proteins can, in turn, induce changes in several biological processes [66], which may be involved in antimetastasis.

Our research uncovered an intriguing finding, DH_31 did not appear to influence the mRNA levels of STAT3 (Figure 6A) but it did affect downstream target genes (Figure 8B). This outcome aligns with prior studies that have demonstrated that compounds binding covalently to cysteine residues in STAT3 can impede its transcriptional activity [13,67]. Notably, DH_31 interacts with the linker domain residues Gly558 and Phe559 (Figure 7C), which are different from those of TM-(–)-18 (Figure 7D) in the linker domain. Effective DNA binding also depends on residues in the linker domain [68,69]. Consequently, any structural perturbations or interactions involving the naphthalene ring could directly influence the binding affinity and specificity of the DNA-binding domain through alterations in the linker region. Our finding implies that the naphthalene ring of DH_31 is essential for binding to STAT3, as it is more effective at stabilizing the interaction than TM-(−)-18 and facilitates the covalent binding of the cyano group to Cys468 in the DNA-binding domain, ultimately leading to a reduction in STAT3 accumulation. Additionally, Cys468 was found to be an important amino acid involved in covalent binding with STAT3 inhibitors, representing a novel site for therapeutic development [40,41].

These findings suggest that DH_31 may play a role in disrupting downstream EMT marker proteins by blocking their functions as a transcription factors, rather than mediating through STAT3 transcriptional levels. DH_31 induces ubiquitin–proteasomal degradation, contributing to the reduction in total STAT3 protein levels. This mechanism may result in the inhibition of the metastasis initiation.

## 4. Materials and Methods

### 4.1. Synthesis of Derivatives of RT Right-Half Analogs

The RT right-half analogs (Figure 1B) were newly synthesized as described in the procedures for the synthesis of DH-17, DH-20, DH-23, DH-26, DH-28, DH-30, and DH-31, and TM-(−)-18 was synthesized according to previous research [27]. The ^1^H-NMR and ^13^C-NMR values are presented in the Supporting Information, Figures S1–S16.

#### Procedures for Synthesis of 2a–3f

**Scheme 1:**
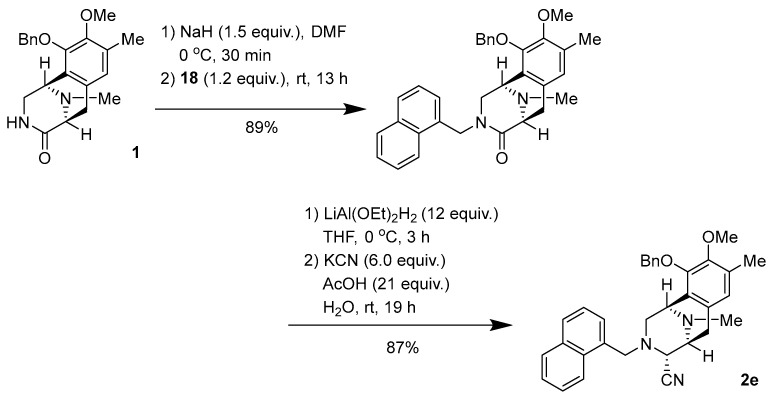
Synthesis of **2e**.

Synthesis of (1R,4R,5S)-10-(benzyloxy)-9-methoxy-8,11-dimethyl-3-(naphthalen-1-ylmethyl)-1,2,3,4,5,6-hexahydro-1,5-epiminobenzo[d]azocine-4-carbonitrile (2e) (Scheme 1).

To a solution of NaH (60% oil dispersion, 17.6 mg, 440 µmol) in DMF (2.0 mL), lactam **1** (104 mg, 297 µmol) in DMF (2.0 mL) was slowly added over 10 min at 0 °C. The reaction mixture was stirred for 30 min at 0 °C, after which a solution of bromide (84.3 mg, 355 µmol, 1.2 equiv.) in DMF (2.0 mL) was added dropwise over 10 min. The reaction mixture was stirred for 13 h at 25 °C. The reaction mixture was diluted with H_2_O (60 mL) and a saturated potassium sodium tartrate solution (40 mL), and extracted with CHCl_3_ (3 × 60 mL). The combined extracts were washed with brine (60 mL), dried over Na_2_SO_4_, and concentrated in vacuo to give a residue. The residue was purified by SiO_2_ flash column chromatography (CHCl_3_:MeOH = 19:1) to afford *N*-alkylated lactam (130 mg, 89%) as a colorless amorphous sample.

To a solution of lactam (51.3 mg, 104 µmol) in THF (2.5 mL) at 0 °C, LiAlH_2_(OEt)_2_ (1.0 mol/L in CH_2_Cl_2_, 1.3 mL, 1.3 mmol, 12 eq.) was slowly added over 4 min. The reaction mixture was stirred at 0 °C for 3 h. The reaction mixture was quenched with AcOH (125 µL, 2.19 mmol, 21 equiv.), followed by the addition of KCN (44.2 mg, 625 µmol, 6.0 equiv.) in H_2_O (225 µL), and stirring was continued for 19 h at 25 °C. The reaction mixture was neutralized with a 5% NaHCO_3_ solution and diluted with saturated Rochell’s salt aq., and the mixture was stirred for 1 h. The reaction mixture was extracted with CHCl_3_ (3 × 40 mL). The combined extracts were washed with brine (40 mL), dried over Na_2_SO_4_, and concentrated in vacuo to give a residue. The residue was purified by SiO_2_ flash column chromatography (CHCl_3_:MeOH = 49:1) to afford compound **2e** (45.7 mg, 87%) as a colorless amorphous sample. ${\left[\alpha \right]}_{D}^{24}$ +21.4 (*c* 0.26, CHCl_3_); IR (KBr) 3019, 2933, 2825, 1483, 1321, 1216, 1158, 1061, 1028, 667, 478, 467, 459, 444, 430, 418, 411, 407 cm^−1^; ^1^H-NMR (400 MHz, CDCl_3_) δ 7.77–7.72 (2H, m, 1′-Np), 7.43 (1H, d, *J* = 8.5 Hz, 1′-Np), 7.41–7.37 (1H, m, 1′-Np), 7.36–7.26 (7H, m, 1′-Np, 10-OBn, overlapped), 7.03–6.99 (1H, m, 1′-Np), 6.53 (1H, s, 7-H), 5.00 (1H, d, *J* = 11.2 Hz, 10-OCH_2_Ph), 4.86 (1H, d, *J* = 11.2 Hz, 10-OCH_2_Ph), 3.97–3.94 (2H, m, *J* = 12.7 Hz, 1-H, 1′-H, overlapped), 3.81 (1H, d, *J* = 12.7 Hz, 1′-H), 3.80 (3H, s, 9-OCH_3_), 3.47 (1H, d, *J* = 2.1 Hz, 4-H), 3.13 (1H, d, *J* = 7.6 Hz, 5-H), 2.94 (1H, dd, *J* = 10.6, 2.9 Hz, 2-H), 2.85 (1H, dd, *J* = 17.7, 7.6 Hz, 6-H), 2.57 (1H, d, *J* = 10.6 Hz, 2-H), 2.34 (3H, s, 8-CH_3_), 2.14 (3H, s, ^11^*N*-CH_3_), 1.95 (1H, d, *J* = 17.7 Hz, 6-H); ^13^C-NMR (100 MHz, CDCl_3_) δ: 148.9 (C), 148.0 (C), 137.5 (C), 133.7 (C), 132.0 (C), 131.8 (C), 130.0 (C), 129.8 (C), 128.7 (CH), 128.5 (CH × 2), 128.5 (CH × 2), 128.2 (CH), 128.0 (CH), 127.8 (CH), 126.4 (C), 125.6 (CH), 125.4 (CH), 125.1 (CH), 124.7 (CH), 124.7 (CH), 116.3 (C), 74.4 (CH_2_), 60.1 (CH_3_), 58.2 (CH_2_), 57.9 (CH), 55.2 (CH), 54.1 (CH_2_), 52.9 (CH), 41.3 (CH_3_), 24.9 (CH_2_), 15.8 (CH_3_); EIMS *m*/*z* (%) 503 (M^+^, 2), 295 (25), 294 (100), 204 (20), 203 (20), 141 (11); HRMS (EI) *m*/*z* 503.2573 (M^+^, calcd for C_33_H_33_N_3_O_2_, 503.2575).

**Scheme 2:**
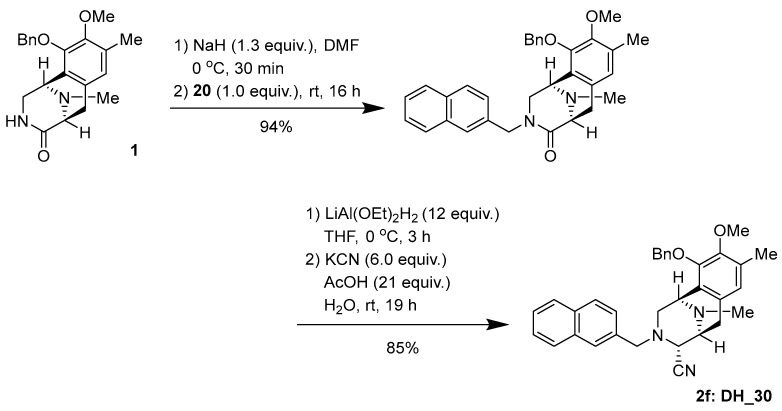
Synthesis of **2f**: DH_30.

Synthesis of (1R,4R,5S)-10-(benzyloxy)-9-methoxy-8,11-dimethyl-3-(naphthalen-2-ylmethyl)-1,2,3,4,5,6-hexahydro-1,5-epiminobenzo[d]azocine-4-carbonitrile (2f: DH_30) (Scheme 2).

To a solution of NaH (60% oil dispersion, 7.8 mg, 195 µmol) in DMF (1.0 mL), lactam **1** (54.5 mg, 155 µmol) in DMF (1.0 mL) was slowly added over 10 min at 0 °C. The reaction mixture was stirred for 30 min at 0 °C, after which a solution of bromide (37.9 mg, 155 µmol, 1.2 equiv.) in DMF (1.0 mL) was added dropwise over 10 min. The reaction mixture was stirred for 16 h at 25 °C. The reaction mixture was diluted with H_2_O (30 mL) and a saturated potassium sodium tartrate solution (20 mL), and extracted with CHCl_3_ (3 × 30 mL). The combined extracts were washed with brine (30 mL), dried over Na_2_SO_4_, and concentrated in vacuo to give a residue. The residue was purified by SiO_2_ flash column chromatography (CHCl_3_:MeOH = 49:1) to afford *N*-alkylated lactam (71.4 mg, 94%) as a colorless gummy oil.

To a solution of lactam (67.4 mg, 137 µmol) in THF (3.3 mL) at 0 °C, LiAlH_2_(OEt)_2_ (1.0 mol/L in CH_2_Cl_2_, 1.6 mL, 1.6 mmol, 12 eq.) was slowly added over 25 min. The reaction mixture was stirred at 0 °C for 3 h. The reaction mixture was quenched with AcOH (165 µL, 2.87 mmol, 21 equiv.), followed by the addition of KCN (58.5 mg, 821 µmol, 6.0 equiv.) in H_2_O (300 µL), and stirring was continued for 19 h at 25 °C. The reaction mixture was neutralized with a 5% NaHCO_3_ solution and diluted with saturated Rochell’s salt aq., and the mixture was stirred for 1 h. The reaction mixture was extracted with CHCl_3_ (3 × 40 mL). The combined extracts were washed with brine (40 mL), dried over Na_2_SO_4_, and concentrated in vacuo to give a residue. The residue was purified by SiO_2_ flash column chromatography (CHCl_3_:MeOH = 49:1) to afford compound **2f** (58.9 mg, 85%) as a colorless amorphous sample. ${\left[\alpha \right]}_{D}^{24}$ −103.2 (*c* 0.79, CHCl_3_); IR (KBr) 3017, 2938, 2825, 1726, 1602, 1483, 1445, 1322, 1229, 1161, 1063, 901, 858, 822, 745, 700, 668, 479, 419, 411 cm^−1^; ^1^H-NMR (400 MHz, CDCl_3_) δ: 7.76 (1H, m, 1′-Np), 7.63 (1H, d, *J* = 8.7 Hz, 1′-Np), 7.61–7.58 (1H, m, 1′-Np), 7.46–7.39 (2H, m 1′-Np), 7.36 (1H, s, 1′-Np), 7.29–7.26 (3H, m, 10-OBn), 7.20–7.18 (2H, m, 10-OBn), 6.99 (1H, dd, *J* = 8.5, 1.6 Hz, 1′-Np), 6.75 (1H, s, 7-H), 4.99 (1H, d, *J* = 11.4 Hz, 10-OCH_2_Ph), 4.79 (1H, d, *J* = 11.4 Hz, 10-OCH_2_Ph), 3.94 (1H, s, 1-H), 3.78 (3H, s, 9-OCH_3_), 3.69 (3H, m, 4-H, 1′-H, overlapped), 3.23 (1H, d, *J* = 7.4 Hz, 5-H), 3.04 (1H, dd, *J* = 17.6, 7.4 Hz, 6-H), 2.87 (1H, dd, *J* = 11.1, 2.9 Hz, 2-H), 2.53 (1H, dd, *J* = 11.1, 1.0 Hz, 2-H), 2.39 (1H, *J* = 17.6 Hz, 6-H), 2.38 (3H, s, 8-CH_3_), 2.17 (3H, s, ^11^*N*-CH_3_); ^13^C-NMR (100 MHz, CDCl_3_) δ: 148.9 (s, C-9), 148.4 (s, C-10), 137.4 (s, Bn), 134.6 (s, Np), 133.2 (s, Np), 132.8 (s, Np), 130.2 (s, C-6a), 130.1 (s, C-8), 128.4 (d × 2, Bn), 128.3 (d × 2, Bn), 128.0 (d, Bn), 128.0 (CH), 127.7 (CH), 127.5 (CH), 127.0 (CH), 126.6 (s, C-10a), 126.0 (CH), 126.0 (CH), 125.7 (CH), 124.3 (CH), 116.6 (s, 4-CN), 74.4 (CH_2_), 60.0 (CH_3_), 59.3 (CH), 59.0 (CH_2_), 55.4 (CH), 53.5 (CH_2_), 52.8 (CH), 41.3 (CH_3_), 25.1 (CH_2_), 15.9 (CH_3_); EIMS *m*/*z* (%) 503 (M^+^, 2), 295 (27), 294 (100), 204 (22), 203 (19), 141 (10); HRMS (EI) *m*/*z* 503.2573 (M^+^, calcd for C_33_H_33_N_3_O_2_, 503.2575).

**Scheme 3:**
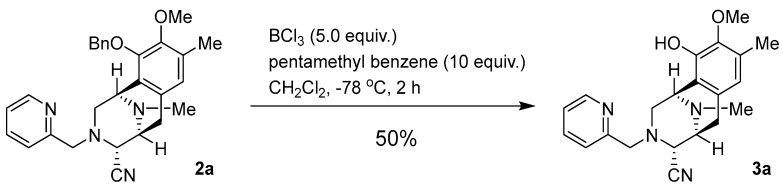
Synthesis of **3a**: DH_17.

Synthesis of (1R,4R,5S)-10-hydroxy-9-methoxy-8,11-dimethyl-3-(pyridin-2-ylmethyl)-1,2,3,4,5,6-hexahydro-1,5-epiminobenzo[d]azocine-4-carbonitrile (3a: DH_17) (Scheme 3).

To a solution of **2a** (41.3 mg, 90.9 μmol) and pentamethylbenzene (138 mg, 909 μmol, 10.0 eq.) in CH_2_Cl_2_ (12.0 mL), BCl_3_ (1.0 mol/L in CH_2_Cl_2_, 455 µL, 455 µmol, 5 eq.) was added over 30 min at −78 °C and the mixture was stirred for 2 h. The reaction mixture was diluted with CH_2_Cl_2_ (30.0 mL) and quenched with a saturated NaHCO_3_ solution at 0 °C. The mixture was extracted with CH_2_Cl_2_ (3 × 30 mL). The combined extracts were washed with brine (30 mL), dried over Na_2_SO_4_ and concentrated in vacuo to give a residue. The residue was purified by SiO_2_ flash column chromatography (CHCl_3_:MeOH = 19:1) to afford compound **3a** (16.4 mg, 50%) as a colorless amorphous sample. ${\left[\alpha \right]}_{D}^{24}$ −82.8 (*c* 0.09, CHCl_3_); IR (KBr) 3534, 3020, 2941, 2829, 2399, 1592, 1432, 1216, 1168, 1060, 1028, 755, 669, 432, 419, 407 cm^−1^; ^1^H-NMR (400 MHz, CDCl_3_) δ 8.45 (1H, dt, *J* = 4.9, 0.9 Hz, 4′-H), 7.35 (1H, td, *J* = 7.9, 1.8 Hz, 6′-H), 7.08–7.05 (1H, m, 5′-H), 6.56 (1H, d, *J* = 7.9 Hz, 7′-H), 6.49 (1H, s, 7-H), 5.88 (1H, brs, 10-OH), 4.08 (1H, s, 1-H), 3.78 (1H, d, *J* = 1.8 Hz, 4-H), 3.76 (2H, s, 1′-H), 3.74 (3H, s, 9-OCH_3_), 3.32 (1H, d, *J* = 7.4 Hz, 5-H), 3.12 (1H, dd, *J* = 17.3, 7.4 Hz, 6-H), 3.02 (1H, dd, *J* = 11.0, 2.7 Hz, 2-H), 2.69 (1H, dt, *J* = 11.0, 1.0 Hz, 2-H), 2.44 (3H, s, ^11^*N*-CH_3_), 2.42 (1H, d, *J* = 17.3 Hz, 6-H), 2.31 (3H, s, 8-CH_3_); ^13^C-NMR (100 MHz, CDCl_3_) δ 157.8 (C), 149.1 (CH), 145.6 (C), 142.9 (C), 136.4 (CH), 130.7 (C), 128.2 (C), 122.1 (CH), 121.9 (CH), 120.4 (CH), 119.5 (C), 116.6 (C), 60.8 (CH_3_), 60.7 (CH_2_), 59.7 (CH), 55.6 (CH), 52.8 (CH_2_), 52.5 (CH), 41.6 (CH_3_), 25.1 (CH_2_), 15.8 (CH_3_); EIMS *m*/*z* (%) 364 (M^+^, 1), 205 (18), 204 (100), 189 (11); HRMS (EI) *m*/*z* 364.1899 (M^+^, calcd for C_21_H_24_N_4_O_2_, 364.1899).

**Scheme 4:**
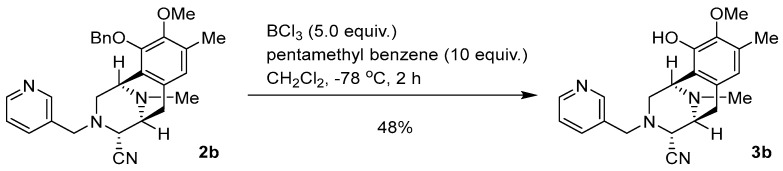
Synthesis of **3b**: DH_20.

Synthesis of (1R,4R,5S)-10-hydroxy-9-methoxy-8,11-dimethyl-3-(pyridin-3-ylmethyl)-1,2,3,4,5,6-hexahydro-1,5-epiminobenzo[d]azocine-4-carbonitrile (3b: DH_20) (Scheme 4).

To a solution of **2b** (44.1 mg, 97.0 μmol) and pentamethylbenzene (152 mg, 970 μmol, 10.0 eq.) in CH_2_Cl_2_ (12.0 mL), BCl_3_ (1.0 mol/L in CH_2_Cl_2_, 485 µL, 485 µmol, 5 eq.) was added over 30 min at −78 °C and the mixture was stirred for 2 h. The reaction mixture was diluted with CH_2_Cl_2_ (30.0 mL) and quenched with a saturated NaHCO_3_ solution at 0 °C. The mixture was extracted with CH_2_Cl_2_ (3 × 30 mL). The combined extracts were washed with brine (30 mL), dried over Na_2_SO_4_ and concentrated in vacuo to give a residue. The residue was purified by SiO_2_ flash column chromatography (CHCl_3_:MeOH = 19:1) to afford compound **3b** (16.9 mg, 48%) as a colorless amorphous sample. ${\left[\alpha \right]}_{D}^{24}$ −76.3 (*c* 0.093, CHCl_3_); IR (KBr) 2931, 2853, 2821, 1722, 1579, 1456, 1418, 1232, 1162, 1028, 805, 751, 712, 487, 450, 420, 412 cm^−1^; ^1^H-NMR (400 MHz, CDCl_3_) δ 8.44 (1H, d, *J* = 3.8 Hz, 5′-H), 8.24 (1H, s, 3′-H), 7.18 (1H, d, *J* = 7.7 Hz, 7′-H), 7.09 (1H, dd, *J* = 7.7, 3.8 Hz, 6′-H), 6.48 (1H, s, 7-H), 4.09 (1H, s, 1-H), 3.78 (3H, s, 9-OCH_3_), 3.67 (1H, s, 4-H), 3.64 (1H, d, *J* = 15.0 Hz, 1′-H), 3.59 (1H, d, *J* = 15.0 Hz, 1′-H), 3.30 (1H, dd, *J* = 7.5, 0.9 Hz, 5-H), 3.09 (1H, dd, *J* = 17.9, 7.5 Hz, 6-H), 2.97 (1H, dd, *J* = 10.9, 2.7 Hz, 2-H), 2.70 (1H, ddd, *J* = 10.9, 2.2, 1.1 Hz, 2-H), 2.38 (3H, s, ^11^*N*-CH_3_), 2.34 (1H, d, *J* = 17.9 Hz, 6-H), 2.31 (3H, s, 8-CH_3_); ^13^C-NMR (100 MHz, CDCl_3_) δ 149.7 (CH), 148.7 (CH), 145.5 (C), 142.9 (C), 136.1 (CH), 132.7 (C), 130.5 (C), 128.3 (C), 123.4 (CH), 120.4 (CH), 119.2 (C), 116.3 (C), 60.8 (CH_3_), 58.9 (CH), 56.5 (CH_2_), 55.4 (CH), 53.0 (CH_2_), 52.4 (CH), 41.5 (CH_3_), 25.1 (CH_2_), 15.8 (CH_3_); EIMS *m*/*z* (%) 364 (M^+^, 1), 205 (21), 204 (100), 189 (11); HRMS (EI) *m*/*z* 364.1903 (M^+^, calcd for C_21_H_24_N_4_O_2_, 364.1899).

**Scheme 5:**
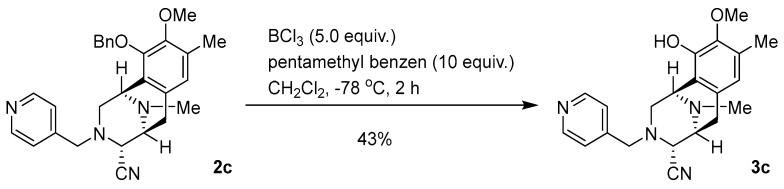
Synthesis of **3c**: DH_23.

Synthesis of (1R,4R,5S)-10-hydroxy-9-methoxy-8,11-dimethyl-3-(pyridin-4-ylmethyl)-1,2,3,4,5,6-hexahydro-1,5-epiminobenzo[d]azocine-4-carbonitrile (3c: DH_23) (Scheme 5).

To a solution of **2c** (85.1 mg, 187 μmol) and pentamethylbenzene (278 mg, 1.87 mmol, 10.0 eq.) in CH_2_Cl_2_ (24.0 mL), BCl_3_ (1.0 mol/L in CH_2_Cl_2_, 936 µL, 936 µmol, 5 eq.) was added over 30 min at −78 °C and the mixture was stirred for 2 h. The reaction mixture was diluted with CH_2_Cl_2_ (30.0 mL) and quenched with a saturated NaHCO_3_ solution at 0 °C. The mixture was extracted with CH_2_Cl_2_ (3 × 100 mL). The combined extracts were washed with brine (100 mL), dried over Na_2_SO_4_ and concentrated in vacuo to give a residue. The residue was purified by SiO_2_ flash column chromatography (CHCl_3_:MeOH = 19:1) to afford compound **3c** (29.3 mg, 43%) as a pale red amorphous sample. ${\left[\alpha \right]}_{D}^{24}$ −73.9 (*c* 0.42, CHCl_3_)

IR (KBr) 3017, 2935, 2821, 1606, 1418, 1319, 1233, 1166, 1061, 1029, 1009, 811, 755, 667, 488, 420, 416, 409 cm^−1^; ^1^H-NMR (400 MHz, CDCl_3_) δ 8.36–8.35 (2H, m, 4′-H, 6′-H), 6.78–6.77 (2H, m, 3′-H, 7′-H), 6.52 (1H, s, 7-H), 4.08 (1H, s, 1-H), 3.78 (3H, s, 9-OCH_3_), 3.71 (1H, s, 4-H), 3.60 (2H, s, 1′-H), 3.32 (1H, d, *J* = 7.5 Hz, 5-H), 3.14 (1H, dd, *J* = 17.6, 7.5 Hz, 6-H), 2.94 (1H, dd, *J* = 10.9, 2.5 Hz, 2-H), 2.65 (1H, d, *J* = 10.9 Hz, 2-H), 2.41 (1H, d, *J* = 17.6 Hz, 6-H), 2.41 (3H, s, ^11^*N*-CH_3_), 2.34 (3H, s, 17.6 Hz, 8-CH_3_); ^13^C-NMR (100 MHz, CDCl_3_) δ 149.7 (CH × 2), 146.7 (C), 145.6 (C), 143.0 (C), 130.6 (C), 128.4 (C), 123.0 (CH × 2), 120.4 (CH), 119.3 (C), 116.4 (C), 60.7 (CH_3_), 59.5 (CH), 57.9 (CH_2_), 55.5 (CH), 52.6 (CH_2_), 52.4 (CH), 41.6 (CH_3_), 25.1 (CH_2_), 15.8 (CH_3_); EIMS *m*/*z* (%) 364 (M^+^, 2), 205 (21), 204 (100), 189 (11); HRMS (EI) *m*/*z*: 364.1900 (M^+^, calcd for C_21_H_24_N_4_O_2_, 364.1899).

**Scheme 6:**
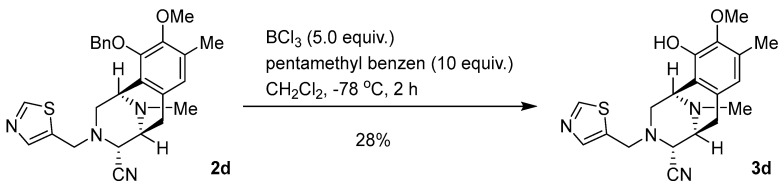
Synthesis of **3d**: DH_26.

Synthesis of (1R,4R,5S)-10-hydroxy-9-methoxy-8,11-dimethyl-3-(thiazol-5-ylmethyl)-1,2,3,4,5,6-hexahydro-1,5-epiminobenzo[d]azocine-4-carbonitrile (3d: DH_26) (Scheme 6).

To a solution of **2d** (31.7 mg, 68.8 μmol) and pentamethylbenzene (104 mg, 688 µmmol, 10.0 eq.) in CH_2_Cl_2_ (12.0 mL), BCl_3_ (1.0 mol/L in CH_2_Cl_2_, 344 µL, 344 µmol, 5 eq.) was added over 30 min at −78 °C and the mixture was stirred for 2 h. The reaction mixture was diluted with CH_2_Cl_2_ (40.0 mL) and quenched with a saturated NaHCO_3_ solution at 0 °C. The mixture was extracted with CH_2_Cl_2_ (3 × 40 mL). The combined extracts were washed with brine (40 mL), dried over Na_2_SO_4_ and concentrated in vacuo to give a residue. The residue was purified by SiO_2_ flash column chromatography (CHCl_3_:MeOH = 19:1) to afford compound **3d** (7.2 mg, 28%) as a colorless oil. ${\left[\alpha \right]}_{D}^{26}$ −28.6 (*c* 0.09, CHCl_3_); IR (KBr) 3020, 1583, 1415, 1216, 1158, 1062, 930, 755, 668, 607, 539, 513, 419, 412 cm^−1^; ^1^H-NMR (400 MHz, CDCl_3_) δ 8.66 (1H, s, 4′-H), 7.61 (1H, s, 6′-H), 6.46 (1H, s, 7-H), 5.63 (1H, brs, 10-OH), 4.08 (1H, s, 1-H), 3.84 (1H, d, *J* = 14.3 Hz, 1′-H), 3.79 (3H, s, 9-OCH_3_), 3.77 (1H, d, *J* = 14.3 Hz, 1′-H), 3.72 (1H, d, *J* = 2.1 Hz, 4-H), 3.29 (1H, d, *J* = 8.0 Hz, 5-H), 3.06 (1H, dd, *J* = 17.5, 8.0 Hz, 6-H), 2.99 (1H, dd, *J* = 10.9, 2.9 Hz, 2-H), 2.76 (1H, ddd, *J* = 10.9, 2.1, 1.1 Hz, 2-H), 2.37 (3H, s, ^11^*N*-CH_3_), 2.35 (1H, d, *J* = 17.5 Hz, 6-H), 2.30 (3H, s, 8-CH_3_); ^13^C-NMR (100 MHz, CDCl_3_) δ 154.0 (CH), 145.4 (s, C-10), 142.8 (s, C-9), 141.8 (CH), 135.7 (s, C-2′), 130.5 (s, C-6a), 128.2 (C), 120.5 (CH), 118.8 (C), 116.1 (C), 60.8 (CH_3_), 58.5 (CH), 55.3 (CH), 53.1 (CH_2_), 52.3 (CH), 51.2 (CH_2_), 41.5 (CH_3_), 24.9 (CH_2_), 15.8 (CH_3_); EIMS *m*/*z* (%) 370 (M^+^, 2), 205 (22), 204 (100), 189 (11); HRMS (EI) *m*/*z*: 370.1463 (M^+^, calcd for C_19_H_22_N_4_O_2_S, 370.1463).

**Scheme 7:**
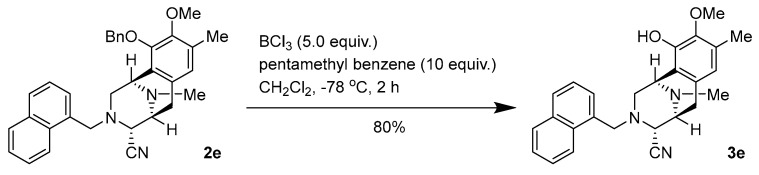
Synthesis of **3e**: DH_28.

Synthesis of (1R,4R,5S)-10-hydroxy-9-methoxy-8,11-dimethyl-3-(naphthalen-1-ylmethyl)-1,2,3,4,5,6-hexahydro-1,5-epiminobenzo[d]azocine-4-carbonitrile (3e: DH_28) (Scheme 7).

To a solution of **2e** (34.1 mg, 67.7 μmol) and pentamethylbenzene (113 mg, 677 µmmol, 10.0 eq.) in CH_2_Cl_2_ (9.0 mL) was added BCl_3_ (1.0 mol/L in CH_2_Cl_2_, 340 µL, 340 µmol, 5 eq.) over 30 min at −78 °C and the mixture was stirred for 2 h. The reaction mixture was diluted with CH_2_Cl_2_ (40.0 mL) and quenched with saturated NaHCO_3_ solution at 0 °C. The mixture was extracted with CH_2_Cl_2_ (3 × 40 mL). The combined extracts were washed with brine (40 mL), dried over Na_2_SO_4_ and concentrated in vacuo to give a residue. The residue was purified by SiO_2_ flash column chromatography (n-Hexane:EtOAc = 2:1) to afford compound **3e** (22.6 mg, 80%) as a colorless amorphous sample. ${\left[\alpha \right]}_{D}^{24}$ −28.6 (*c* 0.09, CHCl_3_); IR (KBr) 3534, 3008, 2939, 2825, 1718, 1587, 1456, 1417, 1297, 1231, 1158, 1059, 1026, 795, 699, 518, 422, 410 cm^−1^; ^1^H-NMR (400 MHz, CDCl_3_) δ 7.77–7.72 (2H, m, 1′-Np-H), 7.48 (1H, d, *J* = 8.2 Hz, 1′-Np-H), 7.40–7.36 (1H, m, 1′-Np-H), 7.35–7.30 (2H, m, 1′-Np-H), 7.03–7.00 (1H, m, 1′-Np-H), 6.31 (1H, s, 7-H), 5.64 (1H, brs, 10-OH), 4.13 (1H, s, 1-H), 4.06 (1H, d, *J* = 12.8 Hz, 1′-H), 3.83 (1H, d, *J* = 12.8 Hz, 1′-H), 3.77 (3H, s, 9-OCH_3_), 3.42 (1H, d, *J* = 2.3 Hz, 4-H), 3.15 (1H, d, *J* = 7.1 Hz, 5-H), 3.09 (1H, dd, *J* = 11.0, 3.0 Hz, 2-H), 2.89–2.83 (2H, m, 2-H, 6-H, overlapped), 2.37 (3H, s, ^11^*N*-CH_3_), 2.34 (3H, s, 8-CH_3_), 1.89 (1H, d, *J* = 17.6 Hz, 6-H); ^13^C-NMR (100 MHz, CDCl_3_) δ 145.2 (C), 142.8 (C), 133.7 (C), 132.0 (C), 131.8 (C), 130.6 (C), 128.7 (CH), 128.0 (CH), 127.9 (CH), 127.8 (C), 125.6 (CH), 125.4 (CH), 125.1 (CH), 124.8 (CH), 120.8 (CH), 119.3 (C), 116.4 (C), 60.8 (CH_3_), 58.2 (CH_2_), 57.5 (CH), 55.2 (CH), 53.7 (CH_2_), 52.6 (CH), 41.6 (CH_3_), 24.8 (CH_2_), 15.8 (CH_3_); EIMS *m*/*z* (%) 413 (M^+^, 2), 205 (21), 204 (100); HRMS (EI) *m*/*z* 413.2105 (M^+^, calcd for C_26_H_27_N_3_O_2_, 413.2103).

**Scheme 8:**
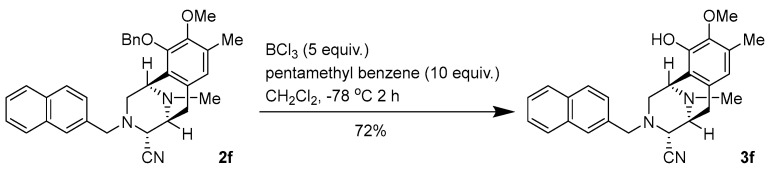
Synthesis of **3f**: DH_31.

Synthesis of (1R,4R,5S)-10-hydroxy-9-methoxy-8,11-dimethyl-3-(naphthalen-2-ylmethyl)-1,2,3,4,5,6-hexahydro-1,5-epiminobenzo[d]azocine-4-carbonitrile (3f: DH_31) (Scheme 8).

To a solution of **2f** (30.4 mg, 60.4 μmol) and pentamethylbenzene (96.1 mg, 604 µmmol, 10.0 eq.) in CH_2_Cl_2_ (8.0 mL), BCl_3_ (1.0 mol/L in CH_2_Cl_2_, 300 µL, 300 µmol, 5 eq.) was added over 30 min at −78 °C and the mixture was stirred for 2 h. The reaction mixture was diluted with CH_2_Cl_2_ (40.0 mL) and quenched with a saturated NaHCO_3_ solution at 0 °C. The mixture was extracted with CH_2_Cl_2_ (3 × 30 mL). The combined extracts were washed with brine (30 mL), dried over Na_2_SO_4_ and concentrated in vacuo to give a residue. The residue was purified by SiO_2_ flash column chromatography (n-Hexane:EtOAc = 2:1) to afford compound **3f** (18.1 mg, 72%) as a colorless amorphous sample. ${\left[\alpha \right]}_{D}^{24}$ −154.1 (*c* 0.09, CHCl_3_); IR (KBr) 3534, 3019, 2940, 2825, 2399, 1730, 1587, 1502, 1458, 1417, 1368, 1318, 1216, 1160, 1059, 1028, 747, 668, 476, 419 cm^−1^; ^1^H-NMR (400 MHz, CDCl_3_) δ 7.77–7.73 (1H, m, 1′-Np-H), 7.65–7.61 (2H, m, 1′-Np-H), 7.46–7.40 (3H, m, 1′-Np-H), 7.03 (1H, dd, *J* = 8.4, 1.7 Hz, 1′-Np-H), 6.53 (1H, s, 7-H), 5.55 (1H, brs, 10-OH), 4.11 (1H, s, 1-H), 3.77 (1H, d, *J* = 13.9 Hz, 1′-H), 3.77 (3H, s, 9-OCH_3_), 3.72 (1H, d, *J* = 13.9 Hz, 1′-H) 3.69 (1H, d, *J* = 2.1 Hz, 4-H), 3.27 (1H, d, *J* = 7.3 Hz, 5-H), 3.11–3.01 (1H, m, 6-H), 3.03 (1H, dd, *J* = 11.6, 2.4 Hz, 2-H), 2.78 (1H, ddd, *J* = 11.6, 2.4, 1.0 Hz, 2-H), 2.39–2.34 (1H, m, 6-H), 2.39 (3H, s, ^11^*N*-CH_3_), 2.37 (3H, s, 8-CH_3_); ^13^C-NMR (100 MHz, CDCl_3_) δ 145.4 (C), 142.8 (C), 134.6 (C), 133.2 (C), 132.8 (C), 130.8 (C), 128.1 (C), 128.0 (CH), 127.7 (CH), 127.5 (CH), 127.0 (CH), 126.1 (CH), 126.0 (CH), 125.7 (CH), 120.4 (CH), 119.4 (C), 116.6 (C), 60.8 (CH_3_), 59.1 (CH_2_), 58.8 (CH), 55.4 (CH), 53.0 (CH_2_), 52.5 (CH), 41.6 (CH_3_), 25.1 (CH_2_), 15.9 (CH_3_); EIMS *m*/*z* (%) 413 (M^+^, 2), 205 (22), 204 (100); HRMS (EI) *m*/*z* 413.2100 (M^+^, calcd for C_26_H_27_N_3_O_2_, 413.2103).

### 4.2. Preparation of Stock Solution

The RT right-half analogs, namely DH_17, DH_20, DH_23, DH_26, DH_28, DH_30, DH_31, and TM-(–)-18 were dissolved in dimethyl sulfoxide (DMSO) to a concentration of 50 mM and were subsequently stored at −20 °C. The final concentration of DMSO for all conditions was less than 0.2% *v*/*v*. No cytotoxicity was observed.

### 4.3. Cell Lines and Reagents

The NSCLC cell lines used in the experiments were obtained from the American Type Culture Collection (Manassas, VA, USA), including NCI-H292 [H292] (ATCC^®^ CRL-1848™, RRID: CVCL_0455) and NCI-H460 [H460] (ATCC^®^ HTB-177™, RRID: CVCL_0459). All cells were cultured in Roswell Park Memorial Institute (RPMI) 1640 medium (Gibco, Gaithersburg, MA, USA), supplemented with 10% fetal bovine serum (HyClone™, Logan, UT, USA), 2mM L-glutamine (Gibco, USA), and 100 units /mL of antibiotic-antimycotic (Gibco). Cells were maintained in a humidified incubator containing 5% CO_2_ at 37 °C until they reached 80% confluence before being used in further experiments.

MTT (3-(4,5-dimethylthiazol-2-yl)-2,5-diphenyltetrazolium bromide), and bovine serum albumin (BSA) were purchased from Sigma Chemical, Inc. (St. Louis, MO, USA). HyClone™ Phosphate-buffered saline (PBS; pH 7.4) was purchased from Cytiva (Logan, UT, USA). The protease inhibitor cocktail was purchased from Roche Applied Science (Indianapolis, IN, USA), and the 10 × RIPA lysis buffer was purchased from Merck Millipore (Darmstadt, Germany). The bicinchoninic acid (BCA) protein assay kit was purchased from Pierce Biotechnology (Rockford, IL, USA). The SuperSignal^TM^ West Pico PLUS Chemiluminescent Substrate, and Dynabeads^TM^ protein G were purchased from Thermo Fisher Sciencetific (Waltham, MA, USA). MG-132 (#2194S) and the primary antibodies specific to STAT3 (#4904S), Vimentin (#5741), N-cadherin (#13116), Slug (#9585), Snail (#3879), β-actin (#4970), and ubiquitin (#14049), as well as the secondary antibodies, anti-rabbit (#7074) and anti-mouse (#7076) IgG horseradish peroxidases, were purchased from Cell Signaling Technology (Danvers, MA, USA). Fluorescent secondary antibodies including Alexa Fluor F(ab)2 fragment of 488, 568, and conjugated goat anti-rabbit, were purchased from Life Technologies (Eugene, OR, USA). The primers used in this study were synthesized by Eurofins Genomics (Plantside Drive Louisville, KY, USA).

### 4.4. Cell Viability Assay

MTT assay was conducted to ascertain cell viability in the presence of DH_17, DH_20, DH_23, DH_26, DH_28, DH_30, DH_31, or TM-(–)-18. H292 and H460 cells (1.0 × 10^4^ cells per well) were seeded in 96-well plates. Following overnight incubation, the cells were treated with different concentrations of RT right–half analogs (0–100 μM) for 24 h at 37 °C. Subsequently, cell viability was assessed using MTT assay, following the manufacturers’ protocols. The optical density was measured at 570 nm using a CLARIOStar microplate reader (BMG Labtech, Ortenberg, Germany). The concentration that resulted in a 50% reduction in viability (IC_50_) for each cell line was further estimated by analyzing the dose–response relationship with GraphPad Prism Version 6.01 (GraphPad Software, San Diego, CA, USA).

### 4.5. Wound Healing Assay

Wound healing assay was conducted to assess the effects of DH_31 on migration. H292 and H460 cells (4.0–4.2 × 10^4^ cells per well) were seeded in 96-well plates. Following overnight incubation, wounds were created by scraping the monolayer cells with a 10 μL pipette tip, and non-adherent cells were washed off with PBS. The cells were treated with different concentrations of DH_31 (0–2.5 μM) in the growth medium containing 1% FBS for 72 h at 37 °C. The level of cell migration across the wounded space was observed at indicated time points (0, 24, 48, and 72 h), and images were captured with a 10× objective using an inverted microscope (Olympus IX51 with DP70; OlympusbAmerica Inc., Center valley, PA, USA). The scratched area was analyzed by using ImageJ™ software (Image J 1.52a, Rasband, W., National Institutes of Health, Bethesda, MD, USA). Cell migration was determined by the rate of cells moving towards the scratched area [70].

### 4.6. Measurement of Cell Resistance to Anoikis

MTT assay was conducted to ascertain cell viability after treatment with DH-31 under detachment conditions. H292 and H460 cells (5.0 × 10^3^ cells per well) were seeded in ultra-low attachment 96-well plates and treated with different concentrations of DH_31 (0–2.5 μM) at 37 °C. At 0, 6, 12, and 24 h of incubation, the cell suspensions were transferred onto regular cell culture plates in growth medium to facilitate attachment. After 3 h, images of cells that survived anoikis and recovered their adherent growth ability were captured with a 10 × objective using an inverted microscope (Olympus IX51 with DP70; OlympusbAmerica Inc., Center valley, PA, USA). Then, the medium in each well was removed and replaced with 100 µL of MTT solution. Cell viability was assessed using MTT assay, following the manufacturers’ protocols. Anoikis resistance was calculated as a percentage compared with the control.

### 4.7. Database Mining of DH_31 Targets, and NSCLC-Associated Genes

In silico methods were employed to identify potential targets for therapeutic intervention against NSCLC. First, the list of genes in NSCLC were obtained from a previous study [31]. Second, a list of prospective drug targets was collected from the Swiss Target Prediction online tool (http://www.swisstargetprediction.ch/; accessed on 20 February 2024) [32]. A Venn diagram (https://bioinfogp.cnb.csic.es/tools/venny/; accessed on 20 February 2024) was developed to identify overlapping genes. The overlapping targets were analyzed using the ShinyGO 0.80 for biological functional enrichment analysis. Results with a *p*-value ≤ 0.05 were considered significant. Dot plots by the ShinyGO tool were used to visualize the GO terms according to the biological processes [33]. The STRING database was used to create a protein–protein interaction (PPI) network for the overlapping targets between NSCLC targets and DH_31 targets. The parameter was specified as Homo sapiens, with a confidence level threshold set at 0.90 for higher reliability for STRING analysis. In order to identify the top 10 core targets, CytoHubba plugin within Cytoscape V3.9.1 (The Cytoscape Consortium, San Diego, CA, USA) was utilized, with the number of degrees used as the primary parameter for ranking [71].

### 4.8. Immunofluorescence Staining and Confocal Microscopy

Immunofluorescence staining and confocal microscopy were conducted to visualize the subcellular localization and spatial distribution of specific proteins. H460 cells (1.0 × 10^4^ cells per well) were seeded in 96-well plates. Following overnight incubation, the cells were treated with different concentrations of DH_31 (0–2.5 μM) for 24 h at 37 °C. After treatment, the cells were permeabilized with 0.5% Triton-X in PBS for 5 min, followed by the blocking of non-specific proteins with 10% FBS in 0.1% Triton-X PBS for 1 h at room temperature. Subsequently, the cells were exposed to primary antibodies (STAT3, Slug, Snail, Vimentin, N-cadherin, ZO1, and ZEB1) and incubated at 4 °C overnight. The cells were then incubated with Alexa Fluor 488- or 594-conjugated secondary antibodies and Hoechst 33342 for 1 h at room temperature. After washing with PBS, the cells were covered with 50% glycerol. The images were captured by confocal microscopy (Zeiss, Jena, Germany). The fluorescence intensity was measured using ImageJ™ software (Image J 1.52a, Rasband, W., National Institutes of Health, Bethesda, MD, USA).

### 4.9. Western Blot Assay

Western blot analysis was performed to detect and quantify specific proteins in a sample. H460 cells (4.0 × 10^5^ cells per well) were seeded in 6 well plates. Following overnight incubation, the cells were treated with different concentrations of DH_31 (0–2.5 μM) for 24 h at 37 °C. After treatment, the cell lysates were isolated using a 1 × RIPA buffer containing a protease inhibitor cocktail for 45 min at 4 °C. The protein content was determined using the BCA protein assay kit. For the Western blot assay, an equal amount of the denatured proteins from cell lysates was separated by electrophoresis on 10 or 12% SDS-PAGE and transferred to 0.2 µm polyvinylidene difluoride (PVDF) membranes (Bio-Rad Laboratories, Hercules, CA, USA). After the membranes were blocked with 5% skim milk, they were then incubated overnight with specific primary antibodies at 4 °C. Subsequently, the membranes were incubated with HRP-conjugated secondary antibodies for 1 h at room temperature. Protein bands were visualized using SuperSignal^TM^ West Pico PLUS Chemiluminescent Substrate. The protein intensity was analyzed using ImageJ™ software (Image J 1.52a, Rasband, W., National Institutes of Health, Bethesda, MD, USA).

### 4.10. Immunoprecipitation Assay

Immunoprecipitation assay was performed to investigate ubiquitin–proteasomal degradation, shedding light on the regulatory mechanisms involved. H460 cells (4.0 × 10^5^ cells per well) were seeded in 6-well plates. Following overnight incubation, the cells were pretreated with 10 μM of a proteasome inhibitor, MG132, for 1 h to prevent proteasomal degradation, and then were treated with different concentrations of DH_31 (0–2.5 μM) for 6 h. After treatment, the cells were lysed as described above. Immunoprecipitation was carried out using the Dynabeads™ Protein G Immunoprecipitation kit, following the manufacturers’ protocols. The magnetic beads were coated with the rabbit anti-STAT3, then incubated with whole-cell lysate and mixed gently overnight at 4 °C. After washing, the supernatant containing the antibody–antigen complex was used to perform Western blot analysis to detect the ubiquitinated STAT3 protein.

### 4.11. Computational Molecular Docking

AutoDock Vina 1.2.5 was employed to perform the docking of DH_31 into the SH2 domain and the DNA-binding domain of STAT3 obtained from the RCSB Protein Data Bank (PDB) [72] under the code 1BG1 [73]. Preprocessing of the protein involved the removal of co-crystal waters, and hydrogen atoms were integrated using UCSF Chimera 1.17.3 [74]. The three-dimensional coordinates of the DH_31 structure were initially generated using MarvinSketch and further optimized utilizing the B3LYP/6-31G (d,p) basis set within the Gaussian 09 program [75]. The protein and ligand were processed through the AutoDockFR suite to create PDBQT files [76]. The configuration file specified a 25 Å × 25 Å × 25 Å grid size with an exhaustiveness value of 32. For the SH2 domain, the central grid box was positioned at coordinates center_x = 105.088, center_y = 75.824, and center_z = 67.806. Likewise, for the DNA-binding domain, the central grid box was positioned at coordinates center_x = 96.037, center_y = 77.713, and center_z = 38.708. Furthermore, UCSF ChimeraX was employed to visualize the patterns of binding interactions [77].

### 4.12. Quantitative Real-Time PCR

Quantitative Real-time PCR was performed to determine the effect of DH_31 on mRNA levels of STAT3 and EMT-related genes. H460 cells (4.0 × 10^5^ cells per well) were seeded in 6-well plates. Following overnight incubation, the cells were treated with different concentrations of DH_31 (0–2.5 μM)) for 8 h at 37 °C. After treatment, total RNA was isolated using the RNeasy kit (Qiagen, Germany) and subsequently subjected to cDNA synthesis using qScript cDNA Supermix (Quanta bio, Beverly, MA, USA), following the manufacturers’ protocols. Real-time qPCR was performed using Luna Universal qPCR Master Mix (NEB, Ipswich, MA, USA) and the CFX 96 Real-time PCR system (Bio-Rad, Hercules, CA, USA). The expression of each cDNA was normalized to that of β-actin, and the comparative Ct method was used to obtain relative expression levels [78,79,80,81,82]. The primers used for Real-time PCR are shown in the in the Supporting Information, Table S1. The list of primers.

### 4.13. Statistics

Data analysis was performed using R version 4.1.3 and RStudio. Statistical analysis was performed using a one-way ANOVA, followed by Dunnett’s post hoc test at a significance level of *p* < 0.05.

## 5. Conclusions

Our study clearly shows the anticancer properties of DH_31 in NSCLC. DH_31 may inhibit migration and anoikis resistance by reducing the protein expression of STAT3 and its downstream targets. Furthermore, DH_31 mediates STAT3 depletion by enhancing the ubiquitin–proteasomal degradation. These data suggest that the DH_31 that was modified from TM-(–)-18 has potential for therapeutic application in metastatic cancer treatment. Therefore, DH_31 should be considered as a lead compound for the development of STAT3-targeted cancer drugs.

## Data Availability

The datasets used and/or analyzed during the current study are available from the corresponding author upon reasonable request.

## Supplementary Materials

The following supporting information can be downloaded at: https://www.mdpi.com/article/10.3390/md22080370/s1, Figure S1. ^1^H-NMR of 2e in CDCl_3_ (400 MHz); Figure S2. ^13^C-NMR of 2e in CDCl_3_ (100 MHz); Figure S3. ^1^H-NMR of 2f: DH_30 in CDCl3 (400 MHz); Figure S4. ^13^C-NMR of 2f: DH_30 in CDCl_3_ (100 MHz); Figure S5. ^1^H-NMR of 3a: DH_17 in CDCl3 (400 MHz); Figure S6. ^13^C-NMR of 3a: DH_17 in CDCl_3_ (100 MHz); Figure S7. ^1^H-NMR of 3b: DH_20 in CDCl3 (400 MHz); Figure S8. ^13^C-NMR of 3b: DH_20 in CDCl_3_ (100 MHz); Figure S9. ^1^H-NMR of 3c: DH_23 in CDCl_3_ (400 MHz); Figure S10. ^13^C-NMR of 3c: DH_23 in CDCl_3_ (100 MHz); Figure S11. ^1^H-NMR of 3d: DH_26 in CDCl3 (400 MHz); Figure S12. ^13^C-NMR of 3d: DH_26 in CDCl_3_ (100 MHz); Figure S13. ^1^H-NMR of 3e: DH_28 in CDCl_3_ (400 MHz); Figure S14. ^13^C-NMR of 3e: DH_28 in CDCl_3_ (100 MHz); Figure S15. ^1^H-NMR of 3f: DH_31 in CDCl_3_ (400 MHz); Figure S16. ^13^C-NMR of 3f: DH_31 in CDCl_3_ (100 MHz); Table S1. The list of primers; Table S2. The list of 64 genes; Table S3. The top 10 significantly enriched GO term; Figure S17. The original images of Western blot in Figure 3C; Figure S18. The original images of Western blot in Figure 5E; Figure S19. The original images of Western blot in Figure 6B–D.
