# Supplementary material for: A New Renieramycin T Right-Half Analog as a Small Molecule Degrader of STAT3
Source: Mar Drugs. 2024 Aug 14;22(8):370. doi: 10.3390/md22080370 (PMC11355573; doi:10.3390/md22080370)

Supporting Information

A New Renieramycin T Right-Half Analog as A Small Molecule degrader of STAT3

Preeyaphan Phookphan [1, 2], Satapat Racha [1, 3], Masashi Yokoya [4], Zin Zin Ei [1, 2], Daiki Hotta[4], Hongbin Zou [5], Pithi Chanvorachote *, [1, 2, 6]

[1] Center of Excellence in Cancer Cell and Molecular Biology, Faculty of Pharmaceutical Sciences, Chulalongkorn University, Bangkok 10330, Thailand

[2] Department of Pharmacology and Physiology, Faculty of Pharmaceutical Sciences, Chulalongkorn University, Bangkok 10330, Thailand

[3] Interdisciplinary Program in Pharmacology, Graduate School, Chulalongkorn University, Bangkok 10330, Thailand

[4] Department of Pharmaceutical Chemistry, Meiji Pharmaceutical University, 2-522-1, Noshio, Kiyose, Tokyo 204-8588, Japan

[5] College of Pharmaceutical Sciences, Zhejiang University, Hangzhou 310058, China

[6] Faculty of Pharmacy, Silpakorn University, Nakhon Pathom 73000, Thailand

Corresponding Author:

Prof. Dr. Pithi Chanvorachote

E-mail: pithi.c@chula.ac.th. Tel: +662 218 8344.

Table of contents Page

Figure S1. ^{1}H-NMR of 2e in $CDCl_3$ (400 MHz) 3

Figure S2.^{13}C-NMR of 2e in $CDCl_3$ (100 MHz) 4

Figure S3. ^{1}H-NMR of 2f: DH_30 in $CDCl_3$ (400 MHz) 5

Figure S4. ^{13}C-NMR of 2f: DH_30 in $CDCl_3$ (100 MHz) 6

Figure S5. ^{1}H-NMR of 3a: DH_17 in $CDCl_3$ (400 MHz) 7

Figure S6. ^{13}C-NMR of 3a: DH_17 in $CDCl_3$ (100 MHz) 8

Figure S7. ^{1}H-NMR of 3b: DH_20 in $CDCl_3$ (400 MHz) 9

Figure S8.^{13}C-NMR of 3b: DH_20 in $CDCl_3$ (100 MHz) 10

Figure S9.^{1}H-NMR of 3c: DH_23 in $CDCl_3$ (400 MHz) 11

Figure S10.^{13}C-NMR of 3c: DH_23 in $CDCl_3$ (100 MHz) 12

Figure S11.^{1}H-NMR of 3d: DH_26 in $CDCl_3$ (400 MHz) 13

Figure S12.^{13}C-NMR of 3d: DH_26 in $CDCl_3$ (100 MHz) 14

Figure S13.^{1}H-NMR of 3e: DH_28 in $CDCl_3$ (400 MHz) 15

Figure S14.^{13}C-NMR of 3e: DH_28 in $CDCl_3$ (100 MHz) 16

Figure S15.^{1}H-NMR of 3f: DH_31 in $CDCl_3$ (400 MHz) 17

Figure S16.^{13}C-NMR of 3f: DH_31 in $CDCl_3$ (100 MHz) 18

Table S1. The list of primers 19

Table S2. The list of 64 core genes 20

Table S3. The top 10 significantly enriched GO term 27

Figure S17. The original images of Western blot in Figure 3C 30

Figure S18. The original images of Western blot in Figure 5E 32

Figure S19. The original images of Western blot in Figure 6B-6D 33

^{1}H-and ^{13}C-NMR spectrum

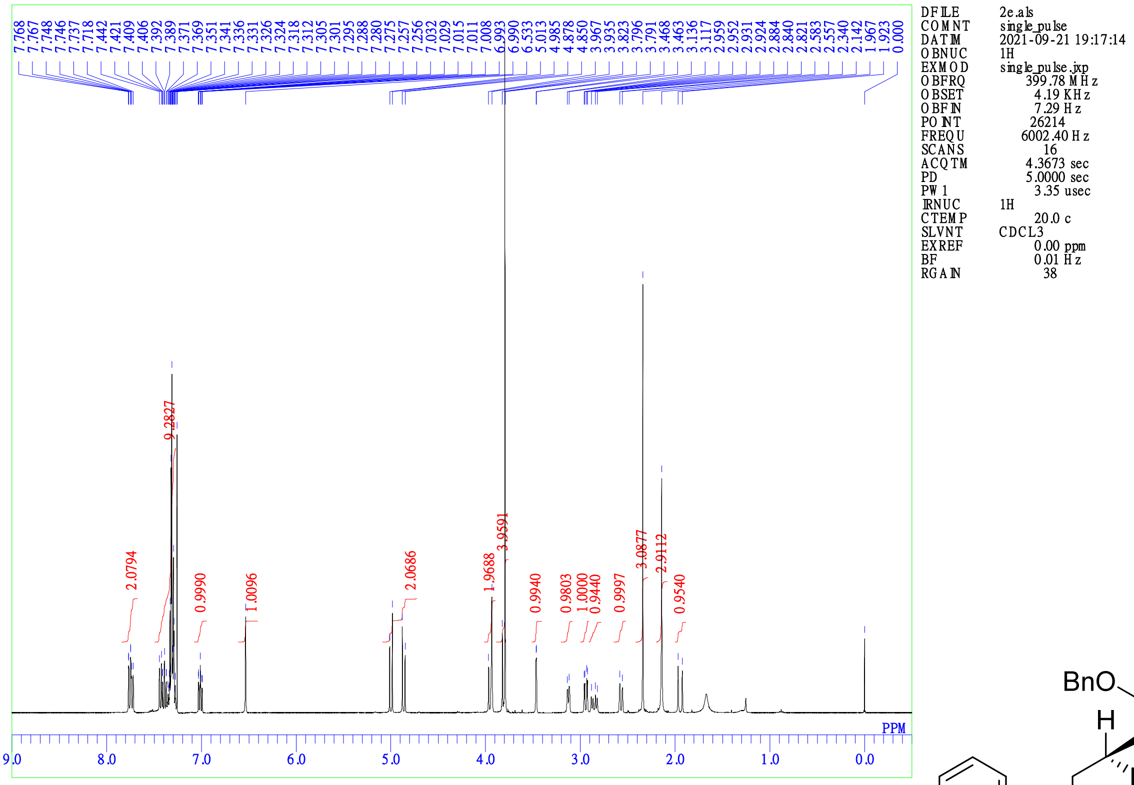

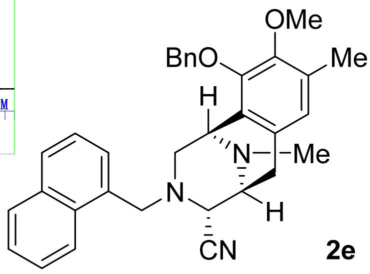

2e

Figure S1. ^{1}H-NMR of 2e in $CDCl_3$ (400 MHz)

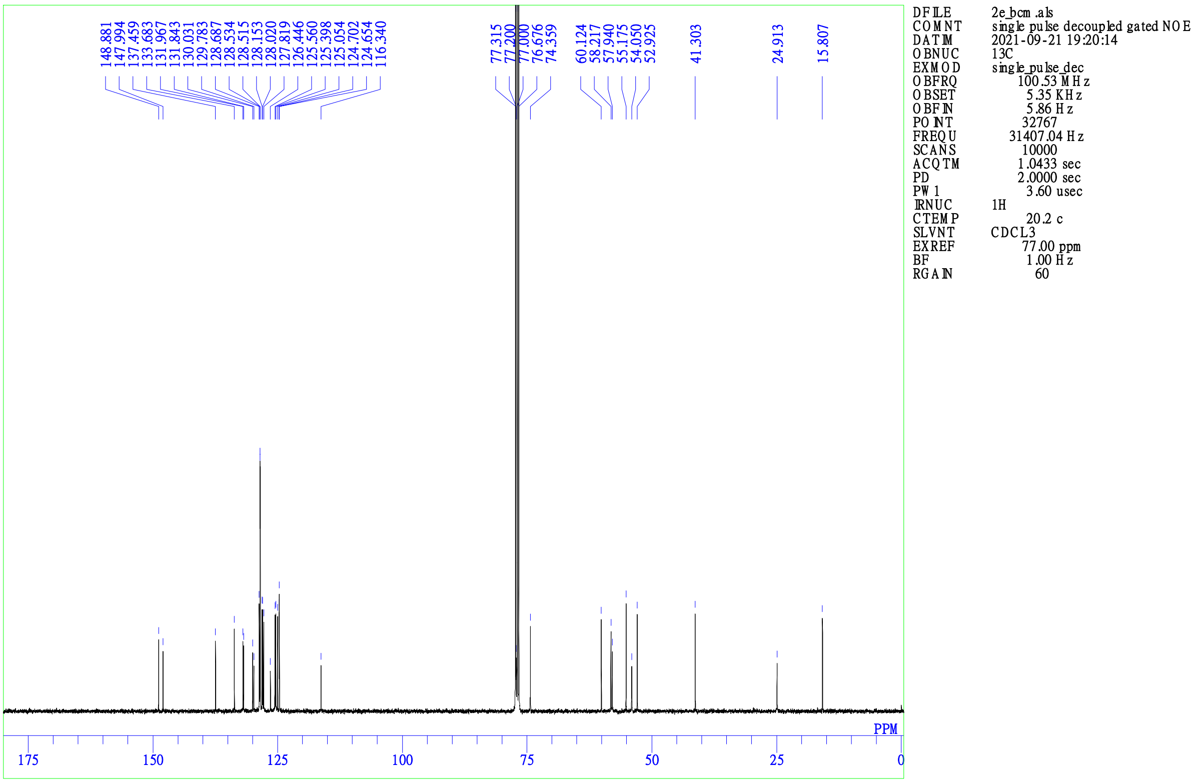

Figure S2.^{13}C-NMR of **2e** in $CDCl_3$ (100 MHz)

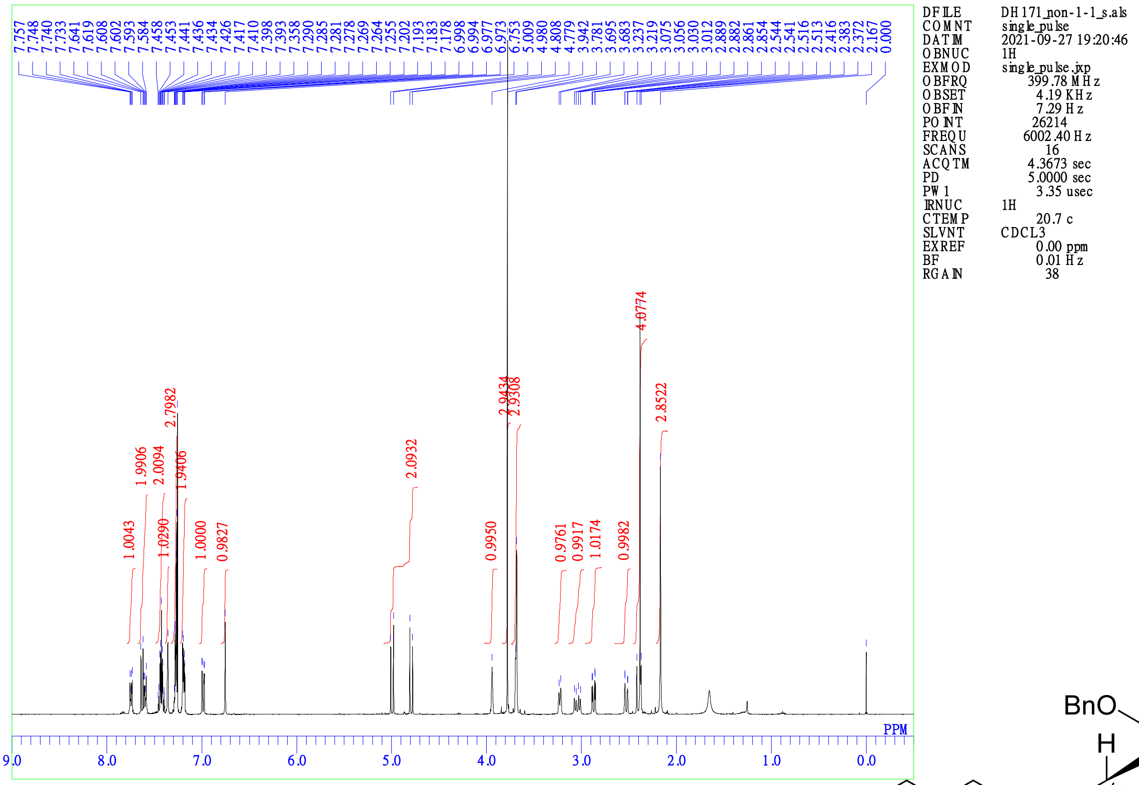

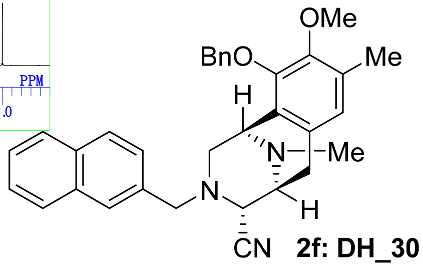

2f: DH_30

Figure S3. ^{1}H-NMR of 2f: DH_30 in $CDCl_3$ (400 MHz)

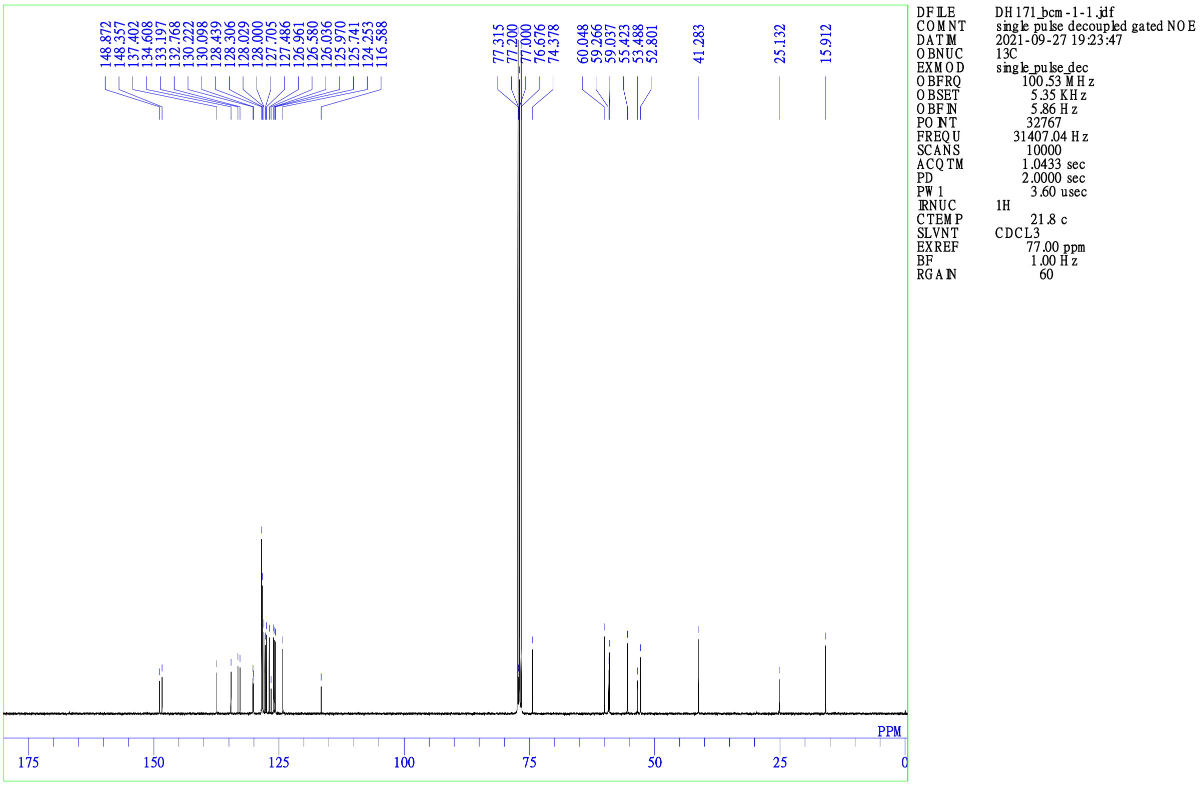

Figure S4. ^{13}C-NMR of 2f: DH_30 in $CDCl_3$ (100 MHz)

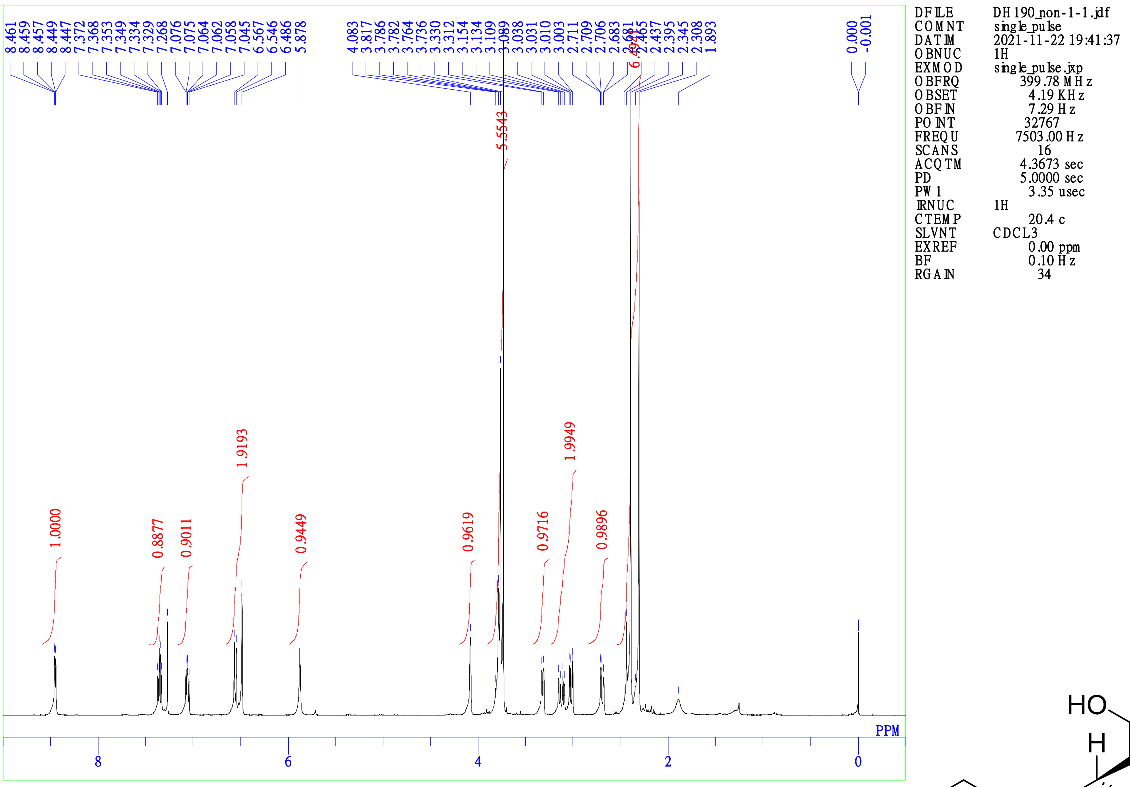

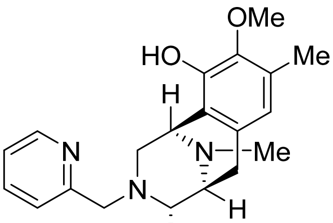

3a

Figure S5. ^{1}H-NMR of 3a: DH_17 in $CDCl_3$ (400 MHz)

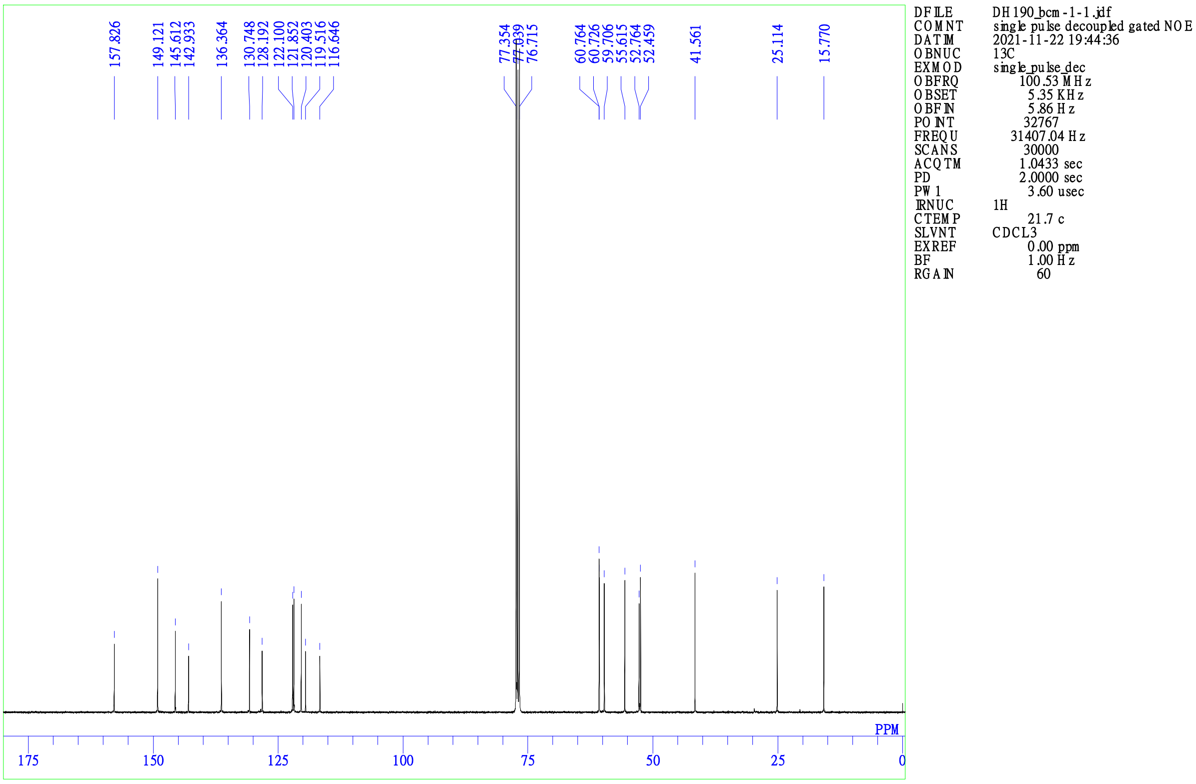

Figure S6. ^{13}C-NMR of 3a: DH_17 in $CDCl_3$ (100 MHz)

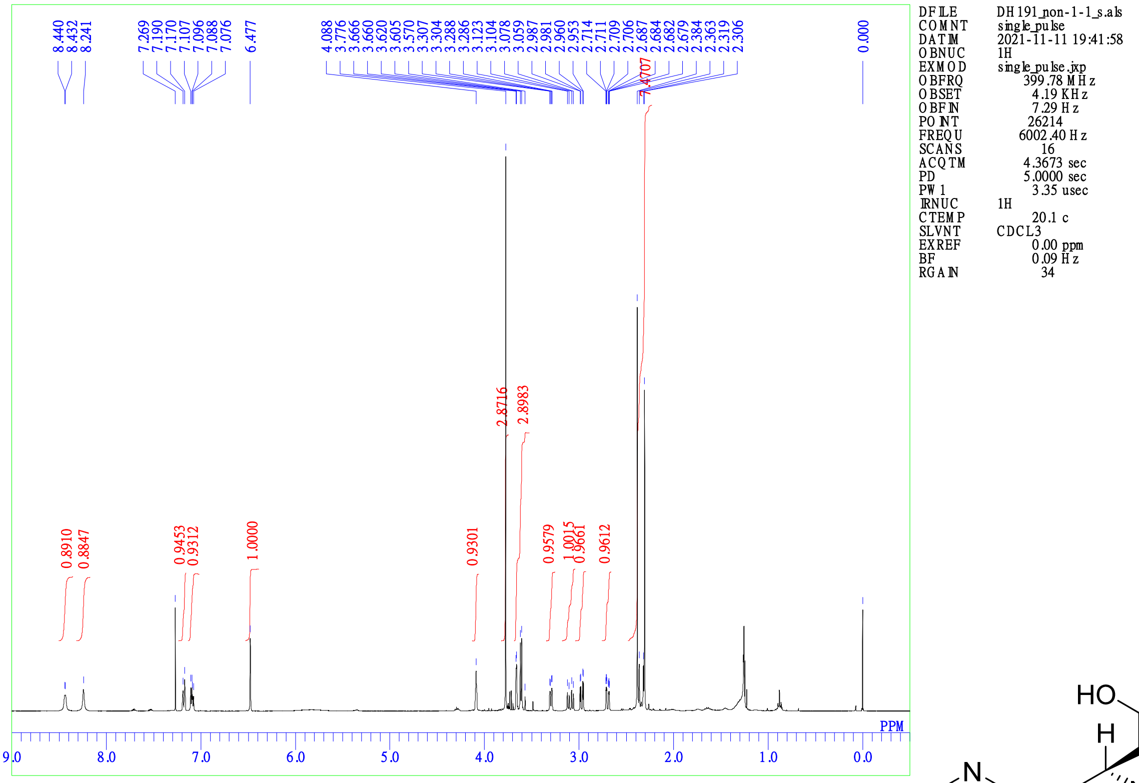

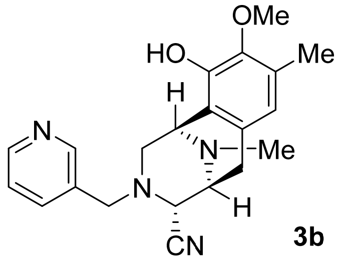

3b

Figure S7. ^{1}H-NMR of 3b: DH_20 in $CDCl_3$ (400 MHz)

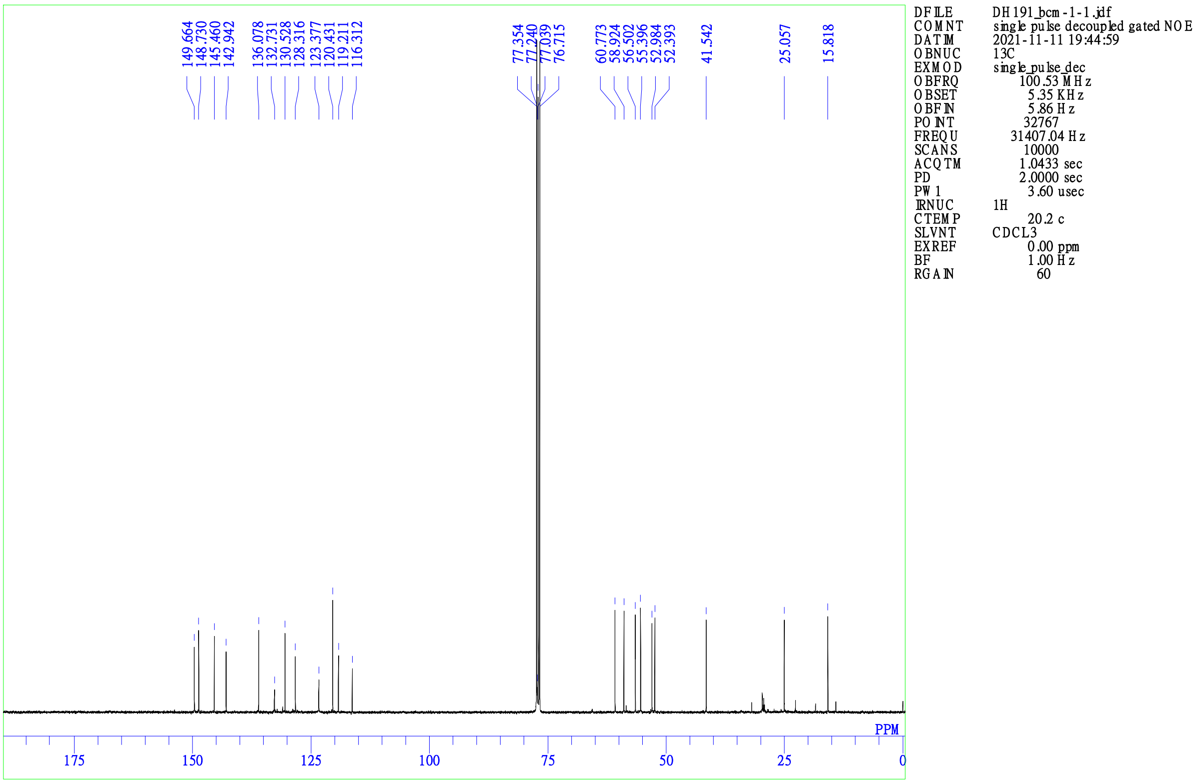

Figure S8. ^{13}C-NMR of 3b: DH_20 in $CDCl_3$ (100 MHz)

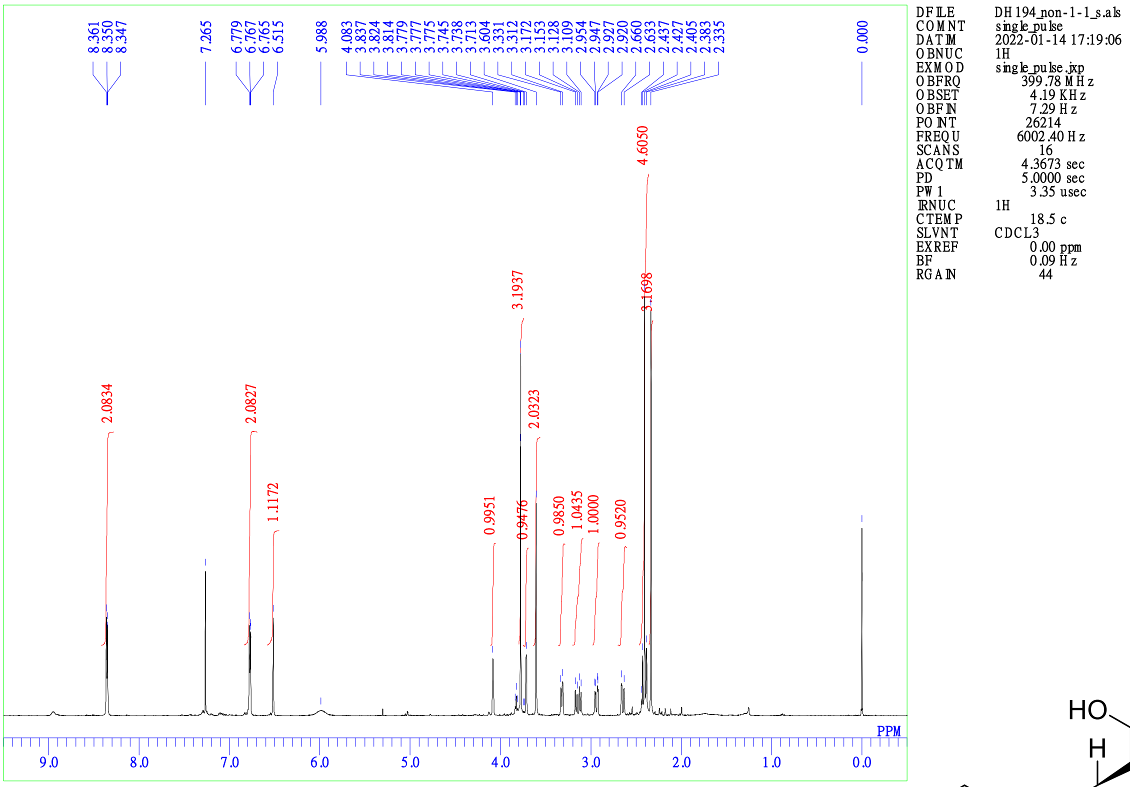

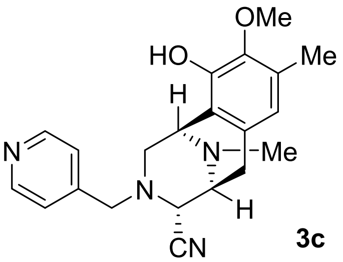

3c

Figure S9. ¹H-NMR of 3c: DH_23 in $CDCl_3$ (400 MHz)

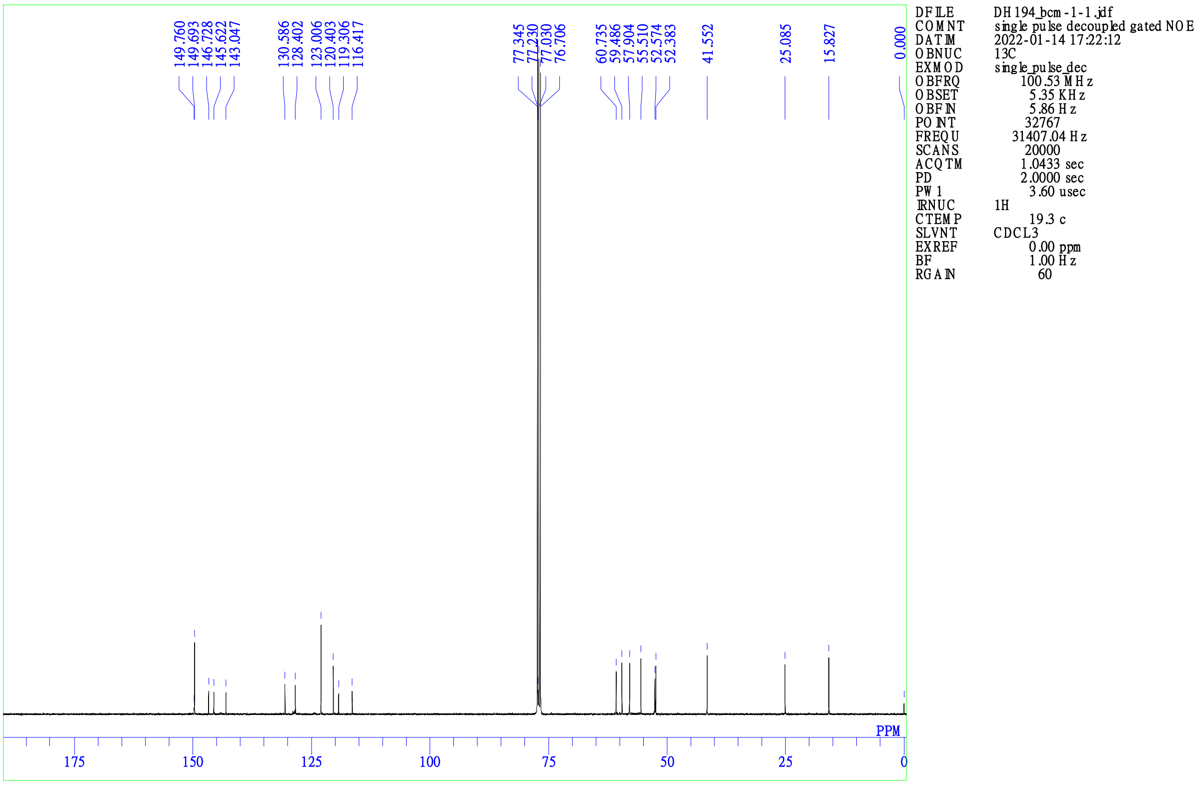

Figure S10. ^{13}C-NMR of 3c: DH_23 in $CDCl_3$ (100 MHz)

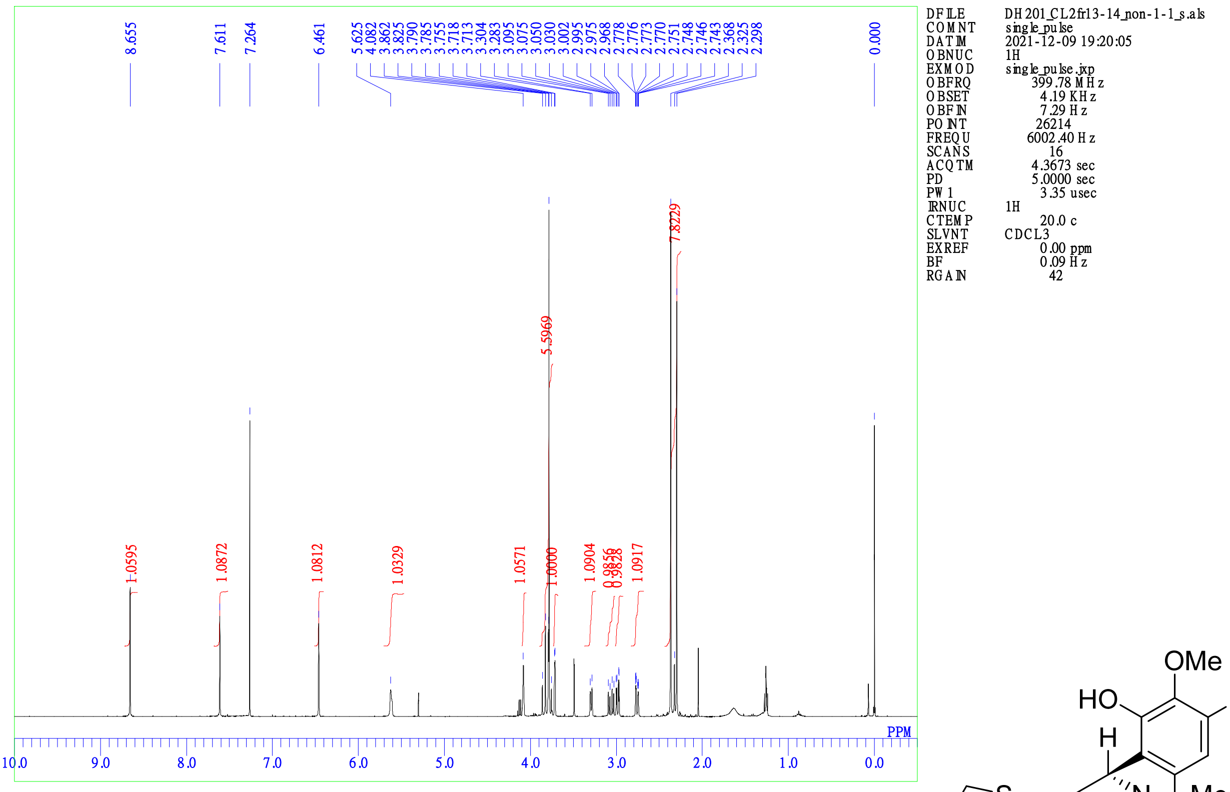

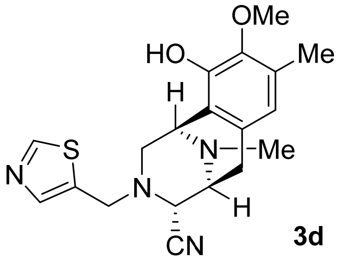

3d

Figure S11.^{1}H-NMR of 3d: DH_26 in $CDCl_3$ (400 MHz)

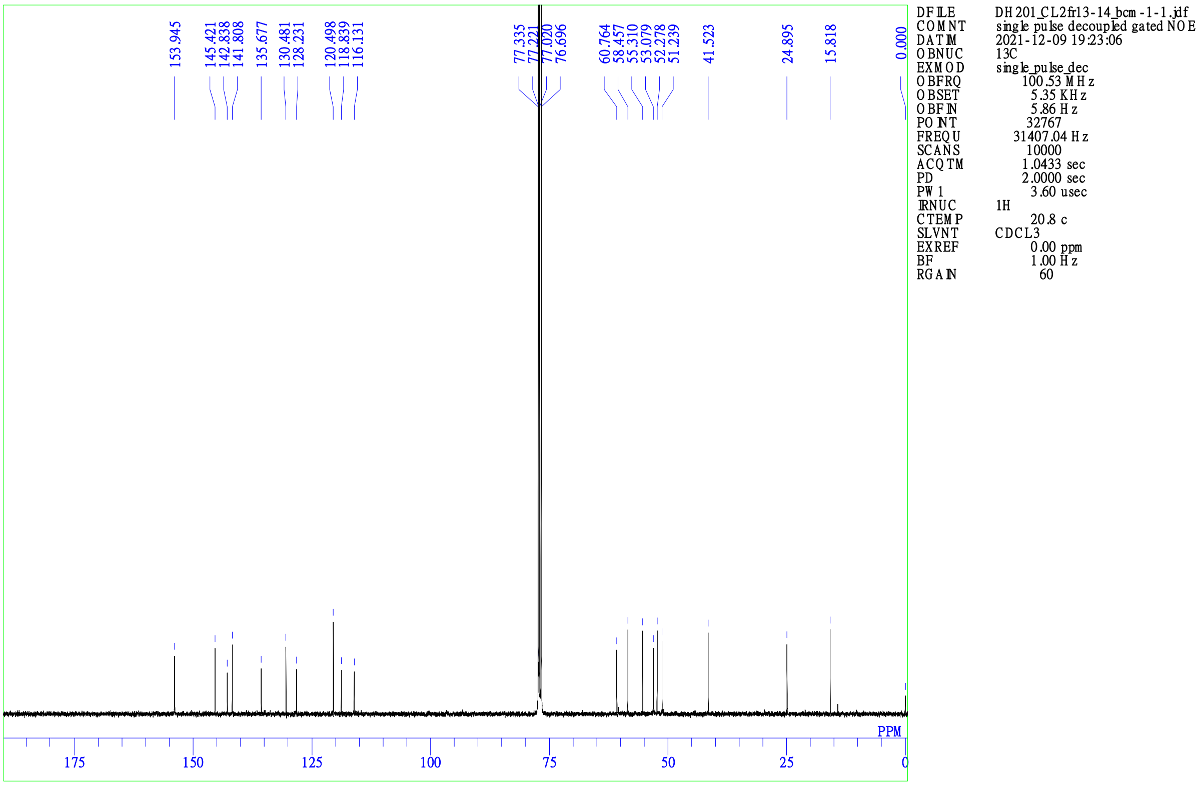

Figure S12. ^{13}C-NMR of 3d: DH_26 in $CDCl_3$ (100 MHz)

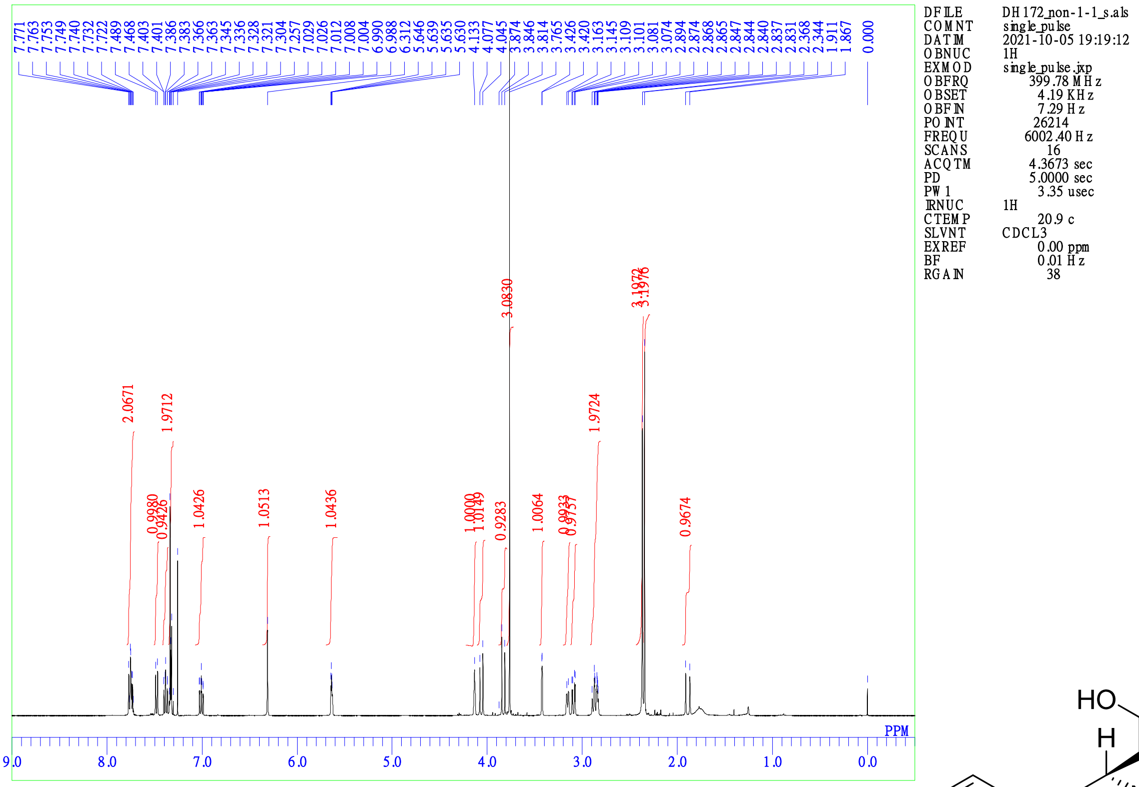

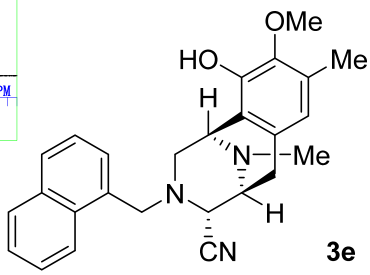

3e

Figure S13. ^{1}H-NMR of 3e: DH_28 in $CDCl_3$ (400 MHz)

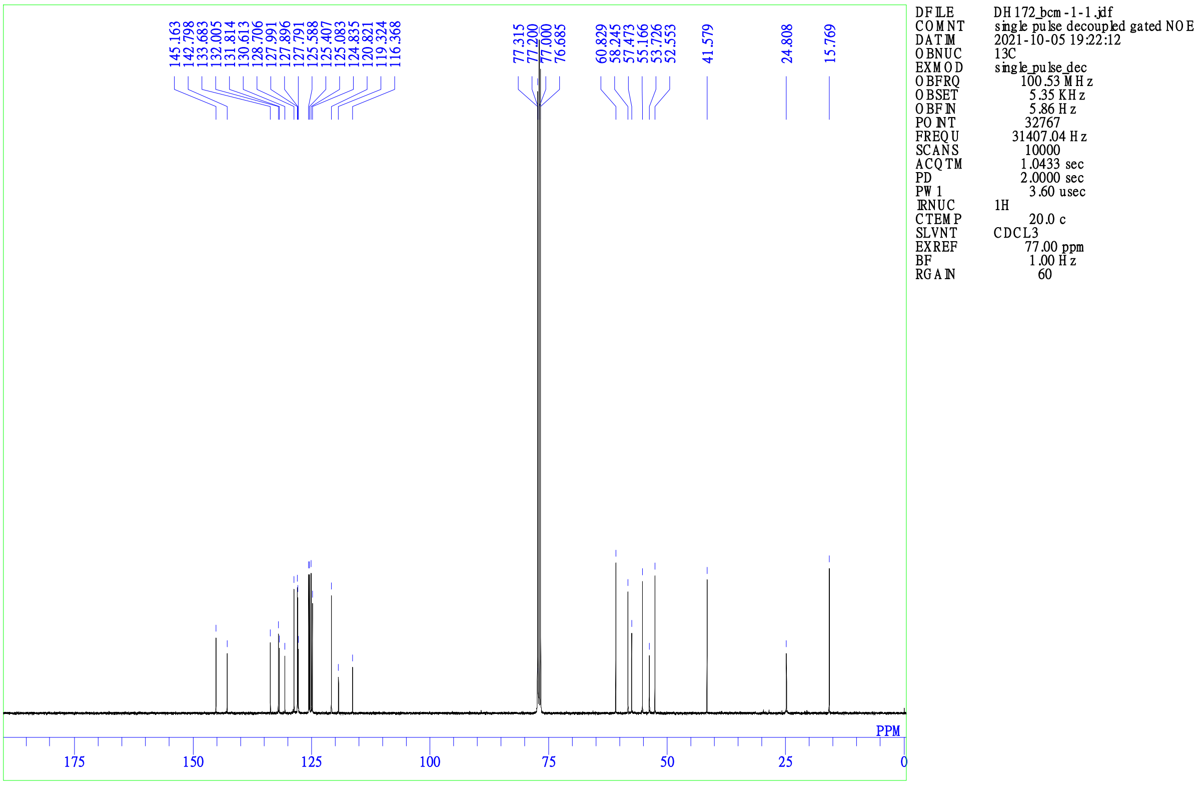

Figure S14.^{13}C-NMR of 3e: DH_28 in $CDCl_3$ (100 MHz)

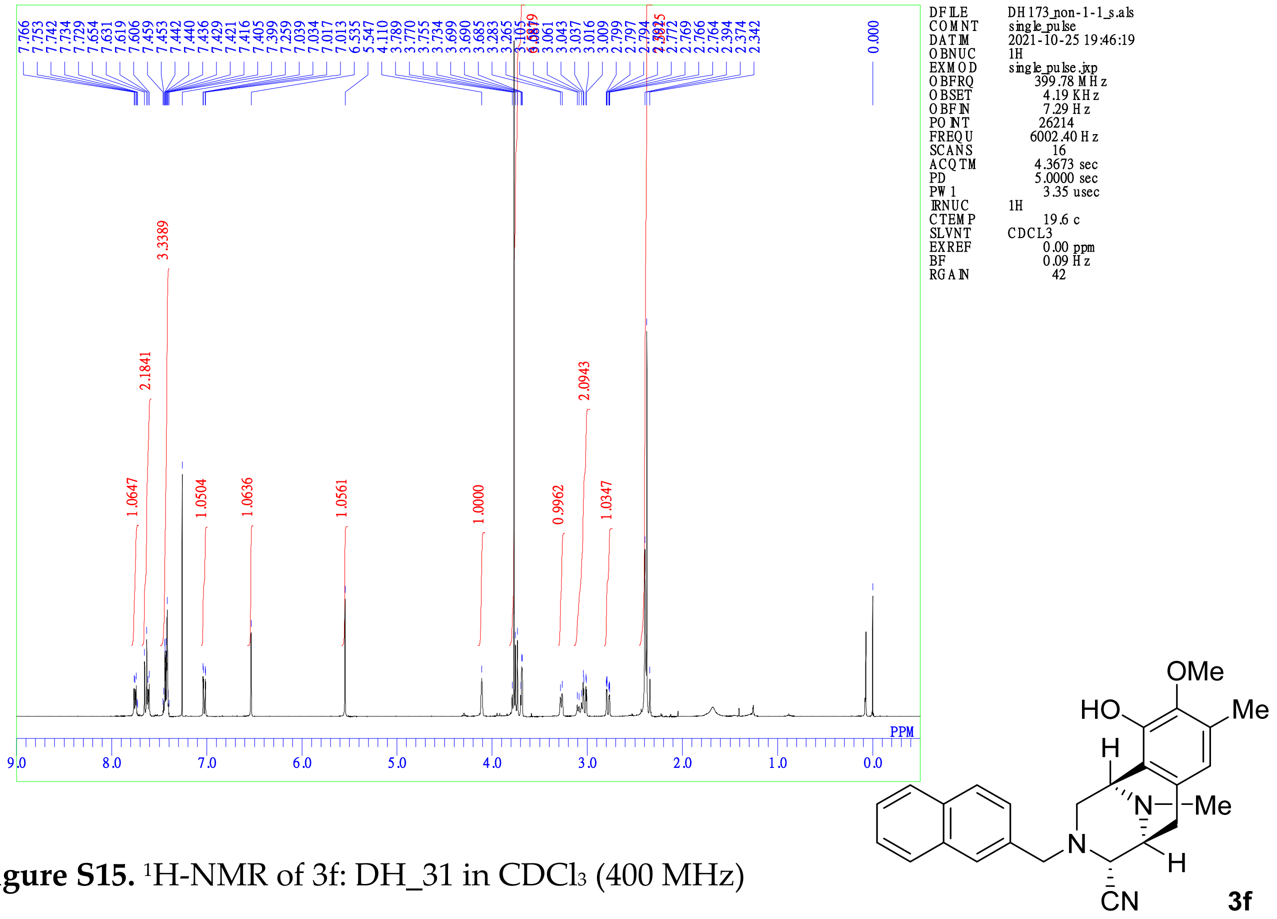

Figure S15. ^{1}H-NMR of 3f: DH_31 in $CDCl_3$ (400 MHz)

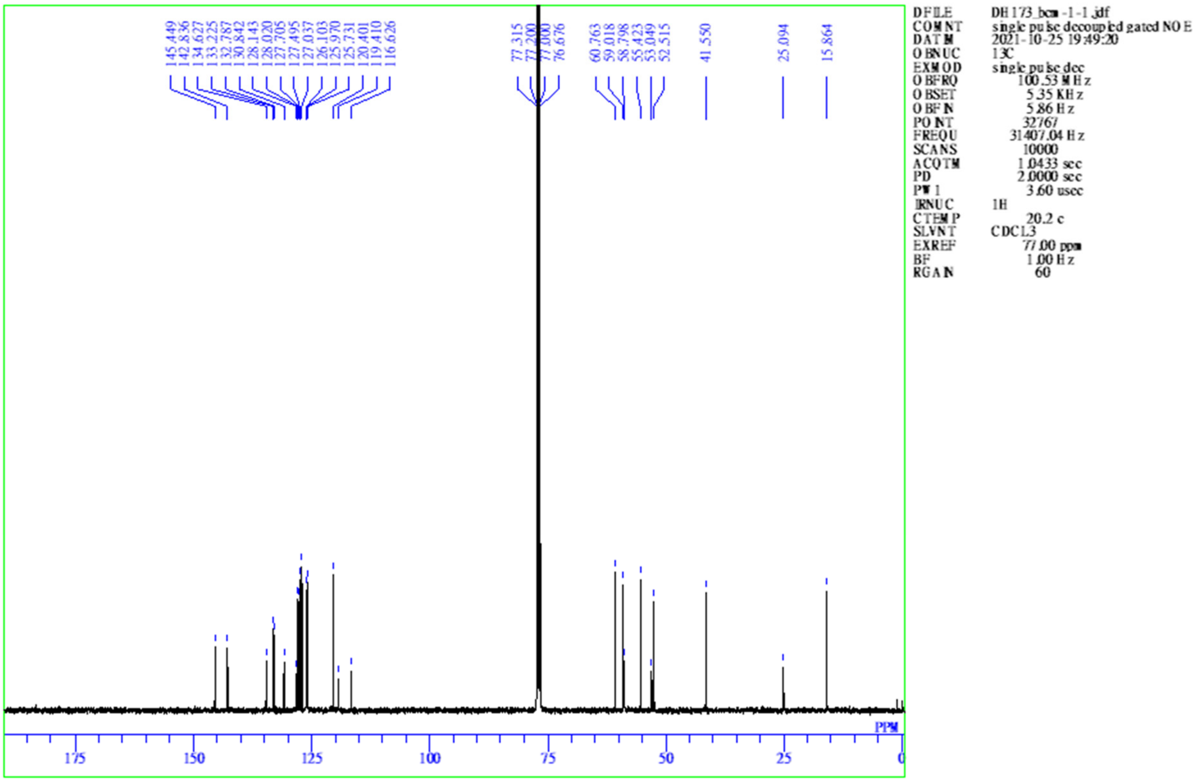

Figure S16. ^{13}C-NMR of 3f: DH_31 in $CDCl_3$ (100 MHz)

Table S1. The list of primers.

Gene	Forward primer (5' to 3')	Reverse primer (5' to 3')	References
ZO1	CAACATACAGTGACGCTTCACA	CACTATTGACGTTTCCCCACTC	[1]
STAT3	CAGAAAGTGTCCTACAAGGGCG	CGTTGTTAGACTCCTCCATGTTC	[2]
Slug	AGCATTTCAACGCCTCCA	GGATCTCTGGTTGTGGTATGAC	[3]
Snail	CTAGCGAGTGGTTCTTCTGC	GTAGTTAGGCTTC CGATTGGG	[3]
Vimentin	ACCCTGCAATCTTTCAGACAG	GATTCCACTTTGCGTTCA AGG	[3]
N-cadherin	GACCGAGAATCACCAAATGTG	GCGTTCCTGTTCCACTCATAG	[3]
β-actin	AGAGCTACGAG CTGCCTGAC	AGCACTGTGTTGGCGTACAG	[4]

Reference

1. Oh, N. S.; Joung, J. Y.; Lee, J. Y.; Kim, Y.; Kim, S. H., Enhancement of Antioxidative and Intestinal Anti-inflammatory Activities of Glycated Milk Casein after Fermentation with Lactobacillus rhamnosus 4B15. *J Agric Food Chem* 2017, 65, (23), 4744-4754.

2. Parisi, C.; Arisi, I.; D'Ambrosi, N.; Storti, A. E.; Brandi, R.; D'Onofrio, M.; Volonte, C., Dysregulated microRNAs in amyotrophic lateral sclerosis microglia modulate genes linked to neuroinflammation. *Cell Death Dis* 2013, 4, (12), e959.

3. Pongrakhananon, V.; Wattanathamsan, O.; Takeichi, M.; Chetprayoon, P.; Chanvorachote, P., Loss of CAMSAP3 promotes EMT via the modification of microtubule-Akt machinery. *J Cell Sci* 2018, 131, (21).

4. Nonpanya, N.; Sanookpan, K.; Sriratanasak, N.; Vinayanuwattikun, C.; Wichadakul, D.; Sritularak, B.; Chanvorachote, P., Artocarpin Targets Focal Adhesion Kinase-Dependent Epithelial to Mesenchymal Transition and Suppresses Migratory-Associated Integrins in Lung Cancer Cells. *Pharmaceutics* 2021, 13, (4).

Table S2. The list of 64 core genes.

initial_alias	converted_alias	description	namespace
EGFR	ENSG00000146648	epidermal growth factor receptor [Source:HGNC Symbol;Acc:HGNC:3236]	ENTREZGENE,GENECARDS,HGNC,UNIPROT_GN,WIKIGENE
MET	ENSG00000105976	MET proto-oncogene, receptor tyrosine kinase [Source:HGNC Symbol;Acc:HGNC:7029]	ENTREZGENE,GENECARDS,HGNC,UNIPROT_GN,WIKIGENE
PIK3CA	ENSG00000121879	phosphatidylinositol-4,5-bisphosphate 3-kinase catalytic subunit alpha [Source:HGNC Symbol;Acc:HGNC:8975]	ENTREZGENE,GENECARDS,HGNC,UNIPROT_GN,WIKIGENE
BRAF	ENSG00000157764	B-Raf proto-oncogene, serine/threonine kinase [Source:HGNC Symbol;Acc:HGNC:1097]	ENTREZGENE,GENECARDS,HGNC,UNIPROT_GN,WIKIGENE
STAT3	ENSG00000168610	signal transducer and activator of transcription 3 [Source:HGNC Symbol;Acc:HGNC:11364]	ENTREZGENE,GENECARDS,HGNC,UNIPROT_GN,WIKIGENE
MTOR	ENSG00000198793	mechanistic target of rapamycin kinase [Source:HGNC Symbol;Acc:HGNC:3942]	ENTREZGENE,GENECARDS,HGNC,UNIPROT_GN,WIKIGENE
PPARG	ENSG00000132170	peroxisome proliferator activated receptor gamma [Source:HGNC Symbol;Acc:HGNC:9236]	ENTREZGENE,GENECARDS,HGNC,UNIPROT_GN,WIKIGENE

JAK3	ENSG00000105639	Janus kinase 3 [Source:HGNC Symbol;Acc:HGNC:6193]	ENTREZGENE,GENECARDS,HGNC,UNIPROT_GN,WIKIGENE
JAK2	ENSG00000096968	Janus kinase 2 [Source:HGNC Symbol;Acc:HGNC:6192]	ENTREZGENE,GENECARDS,HGNC,UNIPROT_GN,WIKIGENE
ABCB1	ENSG00000085563	ATP binding cassette subfamily B member 1 [Source:HGNC Symbol;Acc:HGNC:40]	ENTREZGENE,GENECARDS,HGNC,UNIPROT_GN,WIKIGENE
MMP2	ENSG00000087245	matrix metallopeptidase 2 [Source:HGNC Symbol;Acc:HGNC:7166]	ENTREZGENE,GENECARDS,HGNC,UNIPROT_GN,WIKIGENE
KDR	ENSG00000128052	kinase insert domain receptor [Source:HGNC Symbol;Acc:HGNC:6307]	ENTREZGENE,GENECARDS,HGNC,UNIPROT_GN,WIKIGENE
PTGS2	ENSG00000073756	prostaglandin-endoperoxide synthase 2 [Source:HGNC Symbol;Acc:HGNC:9605]	ENTREZGENE,GENECARDS,HGNC,UNIPROT_GN,WIKIGENE
RAF1	ENSG00000132155	Raf-1 proto-oncogene, serine/threonine kinase [Source:HGNC Symbol;Acc:HGNC:9829]	ENTREZGENE,GENECARDS,HGNC,UNIPROT_GN,WIKIGENE
MAPK8	ENSG00000107643	mitogen-activated protein kinase 8 [Source:HGNC Symbol;Acc:HGNC:6881]	ENTREZGENE,GENECARDS,HGNC,UNIPROT_GN,WIKIGENE
TYMS	ENSG00000176890	thymidylate synthetase [Source:HGNC Symbol;Acc:HGNC:12441]	ENTREZGENE,GENECARDS,HGNC,UNIPROT_GN,WIKIGENE

FLT3	ENSG00000122025	fms related receptor tyrosine kinase 3 [Source:HGNC Symbol;Acc:HGNC:3765]	ENTREZGENE,GENECARDS,HGNC,UNIPROT_GN,WIKIGENE
SLC2A1	ENSG00000117394	solute carrier family 2 member 1 [Source:HGNC Symbol;Acc:HGNC:11005]	ENTREZGENE,GENECARDS,HGNC,UNIPROT_GN,WIKIGENE
HSP90AA1	ENSG00000080824	heat shock protein 90 alpha family class A member 1 [Source:HGNC Symbol;Acc:HGNC:5253]	ENTREZGENE,GENECARDS,HGNC,UNIPROT_GN,WIKIGENE
NTRK1	ENSG00000198400	neurotrophic receptor tyrosine kinase 1 [Source:HGNC Symbol;Acc:HGNC:8031]	ENTREZGENE,GENECARDS,HGNC,UNIPROT_GN,WIKIGENE
PIK3CG	ENSG00000105851	phosphatidylinositol-4,5-bisphosphate 3-kinase catalytic subunit gamma [Source:HGNC Symbol;Acc:HGNC:8978]	ENTREZGENE,GENECARDS,HGNC,UNIPROT_GN,WIKIGENE
MMP14	ENSG00000157227	matrix metallopeptidase 14 [Source:HGNC Symbol;Acc:HGNC:7160]	ENTREZGENE,GENECARDS,HGNC,UNIPROT_GN,WIKIGENE
PLK1	ENSG00000166851	polo like kinase 1 [Source:HGNC Symbol;Acc:HGNC:9077]	ENTREZGENE,GENECARDS,HGNC,UNIPROT_GN,WIKIGENE
MMP7	ENSG00000137673	matrix metallopeptidase 7 [Source:HGNC Symbol;Acc:HGNC:7174]	ENTREZGENE,GENECARDS,HGNC,UNIPROT_GN,WIKIGENE
HDAC1	ENSG00000116478	histone deacetylase 1 [Source:HGNC Symbol;Acc:HGNC:4852]	ENTREZGENE,GENECARDS,HGNC,UNIPROT_GN,WIKIGENE

NOS2	ENSG00000007171	nitric oxide synthase 2 [Source:HGNC Symbol;Acc:HGNC:7873]	ENTREZGENE,GENECARDS,HGNC,UNIPROT_GN,WIKIGENE
ITK	ENSG00000113263	IL2 inducible T cell kinase [Source:HGNC Symbol;Acc:HGNC:6171]	ENTREZGENE,GENECARDS,HGNC,UNIPROT_GN,WIKIGENE
MMP13	ENSG00000137745	matrix metallopeptidase 13 [Source:HGNC Symbol;Acc:HGNC:7159]	ENTREZGENE,GENECARDS,HGNC,UNIPROT_GN,WIKIGENE
NR3C1	ENSG00000113580	nuclear receptor subfamily 3 group C member 1 [Source:HGNC Symbol;Acc:HGNC:7978]	ENTREZGENE,GENECARDS,HGNC,UNIPROT_GN,WIKIGENE
GZMB	ENSG00000100453	granzyme B [Source:HGNC Symbol;Acc:HGNC:4709]	ENTREZGENE,GENECARDS,HGNC,UNIPROT_GN,WIKIGENE
CCKBR	ENSG00000110148	cholecystokinin B receptor [Source:HGNC Symbol;Acc:HGNC:1571]	ENTREZGENE,GENECARDS,HGNC,UNIPROT_GN,WIKIGENE
DPP4	ENSG00000197635	dipeptidyl peptidase 4 [Source:HGNC Symbol;Acc:HGNC:3009]	ENTREZGENE,GENECARDS,HGNC,UNIPROT_GN,WIKIGENE
HSD17B1	ENSG00000108786	hydroxysteroid 17-beta dehydrogenase 1 [Source:HGNC Symbol;Acc:HGNC:5210]	ENTREZGENE,GENECARDS,HGNC,UNIPROT_GN,WIKIGENE
HDAC2	ENSG00000196591	histone deacetylase 2 [Source:HGNC Symbol;Acc:HGNC:4853]	ENTREZGENE,GENECARDS,HGNC,UNIPROT_GN,WIKIGENE
DDX3X	ENSG00000215301	DEAD-box helicase 3 X-linked [Source:HGNC	ENTREZGENE,GENECARDS,HGNC,UNIPROT_GN,WIKIGENE

		Symbol;Acc:HGNC:2745]	
NAMPT	ENSG00000105835	nicotinamide phosphoribosyltransferase [Source:HGNC Symbol;Acc:HGNC:30092]	ENTREZGENE,GENECARDS,HGNC,UNIPROT_GN,WIKIGENE
LDHA	ENSG00000134333	lactate dehydrogenase A [Source:HGNC Symbol;Acc:HGNC:6535]	ENTREZGENE,GENECARDS,HGNC,UNIPROT_GN,WIKIGENE
MAPK10	ENSG00000109339	mitogen-activated protein kinase 10 [Source:HGNC Symbol;Acc:HGNC:6872]	ENTREZGENE,GENECARDS,HGNC,UNIPROT_GN,WIKIGENE
PTGS1	ENSG00000095303	prostaglandin-endoperoxide synthase 1 [Source:HGNC Symbol;Acc:HGNC:9604]	ENTREZGENE,GENECARDS,HGNC,UNIPROT_GN,WIKIGENE
MAPK9	ENSG00000050748	mitogen-activated protein kinase 9 [Source:HGNC Symbol;Acc:HGNC:6886]	ENTREZGENE,GENECARDS,HGNC,UNIPROT_GN,WIKIGENE
WEE1	ENSG00000166483	WEE1 G2 checkpoint kinase [Source:HGNC Symbol;Acc:HGNC:12761]	ENTREZGENE,GENECARDS,HGNC,UNIPROT_GN,WIKIGENE
PLK2	ENSG00000145632	polo like kinase 2 [Source:HGNC Symbol;Acc:HGNC:19699]	ENTREZGENE,GENECARDS,HGNC,UNIPROT_GN,WIKIGENE
ADAM10	ENSG00000137845	ADAM metallopeptidase domain 10 [Source:HGNC Symbol;Acc:HGNC:188]	ENTREZGENE,GENECARDS,HGNC,UNIPROT_GN,WIKIGENE
CDK9	ENSG00000136807	cyclin dependent kinase 9 [Source:HGNC	ENTREZGENE,GENECARDS,HGNC,UNIPROT_GN,WIKIGENE

		Symbol;Acc:HGNC:1780]	
NR3C2	ENSG00000151623	nuclear receptor subfamily 3 group C member 2 [Source:HGNC Symbol;Acc:HGNC:7979]	ENTREZGENE,GENECARDS,HGNC,UNIPROT_GN,WIKIGENE
DRD2	ENSG00000149295	dopamine receptor D2 [Source:HGNC Symbol;Acc:HGNC:3023]	ENTREZGENE,GENECARDS,HGNC,UNIPROT_GN,WIKIGENE
HSP90AB1	ENSG00000096384	heat shock protein 90 alpha family class B member 1 [Source:HGNC Symbol;Acc:HGNC:5258]	ENTREZGENE,GENECARDS,HGNC,UNIPROT_GN,WIKIGENE
ACHE	ENSG00000087085	acetylcholinesterase (Cartwright blood group) [Source:HGNC Symbol;Acc:HGNC:108]	ENTREZGENE,GENECARDS,HGNC,UNIPROT_GN,WIKIGENE
MAP3K5	ENSG00000197442	mitogen-activated protein kinase kinase kinase 5 [Source:HGNC Symbol;Acc:HGNC:6857]	ENTREZGENE,GENECARDS,HGNC,UNIPROT_GN,WIKIGENE
DYRK1A	ENSG00000157540	dual specificity tyrosine phosphorylation regulated kinase 1A [Source:HGNC Symbol;Acc:HGNC:3091]	ENTREZGENE,GENECARDS,HGNC,UNIPROT_GN,WIKIGENE
SLC2A3	ENSG00000059804	solute carrier family 2 member 3 [Source:HGNC Symbol;Acc:HGNC:11007]	ENTREZGENE,GENECARDS,HGNC,UNIPROT_GN,WIKIGENE
PDE5A	ENSG00000138735	phosphodiesterase 5A [Source:HGNC Symbol;Acc:HGNC:8784]	ENTREZGENE,GENECARDS,HGNC,UNIPROT_GN,WIKIGENE

PDK1	ENSG00000152256	pyruvate dehydrogenase kinase 1 [Source:HGNC Symbol;Acc:HGNC:8809]	ENTREZGENE,GENECARDS,HGNC,UNIPROT_GN,WIKIGENE
ADORA2A	ENSG00000128271	adenosine A2a receptor [Source:HGNC Symbol;Acc:HGNC:263]	ENTREZGENE,GENECARDS,HGNC,UNIPROT_GN,WIKIGENE
S1PR3	ENSG00000213694	sphingosine-1-phosphate receptor 3 [Source:HGNC Symbol;Acc:HGNC:3167]	ENTREZGENE,GENECARDS,HGNC,UNIPROT_GN,WIKIGENE
CAPN2	ENSG00000162909	calpain 2 [Source:HGNC Symbol;Acc:HGNC:1479]	ENTREZGENE,GENECARDS,HGNC,UNIPROT_GN,WIKIGENE
FPR1	ENSG00000171051	formyl peptide receptor 1 [Source:HGNC Symbol;Acc:HGNC:3826]	ENTREZGENE,GENECARDS,HGNC,UNIPROT_GN,WIKIGENE
KCNA5	ENSG00000130037	potassium voltage-gated channel subfamily A member 5 [Source:HGNC Symbol;Acc:HGNC:6224]	ENTREZGENE,GENECARDS,HGNC,UNIPROT_GN,WIKIGENE
DRD4	ENSG00000069696	dopamine receptor D4 [Source:HGNC Symbol;Acc:HGNC:3025]	ENTREZGENE,GENECARDS,HGNC,UNIPROT_GN,WIKIGENE
FAAH	ENSG00000117480	fatty acid amide hydrolase [Source:HGNC Symbol;Acc:HGNC:3553]	ENTREZGENE,GENECARDS,HGNC,UNIPROT_GN,WIKIGENE
DYRK1B	ENSG00000105204	dual specificity tyrosine phosphorylation regulated kinase 1B [Source:HGNC	ENTREZGENE,GENECARDS,HGNC,UNIPROT_GN,WIKIGENE

		Symbol;Acc:HGNC: 3092]	
SIGMAR1	ENSG00000147955	sigma non-opioid intracellular receptor 1 [Source:HGNC Symbol;Acc:HGNC: 8157]	ENTREZGENE,GENECARDS,HGNC,UNIPROT_GN,WIKIGENE
PDE2A	ENSG00000186642	phosphodiesterase 2A [Source:HGNC Symbol;Acc:HGNC: 8777]	ENTREZGENE,GENECARDS,HGNC,UNIPROT_GN,WIKIGENE
CRHR1	ENSG00000120088	corticotropin releasing hormone receptor 1 [Source:HGNC Symbol;Acc:HGNC: 2357]	ENTREZGENE,GENECARDS,HGNC,UNIPROT_GN,WIKIGENE

Table S3. The top 10 significantly enriched GO term.

Enrichment FDR	nGenes	Pathway Genes	Fold Enrichment	Pathway	URL	Genes
1.45E-18	38	1994	5.83754	GO:0016310 phosphorylation	http://amigo.geneontology.org/amigo/term/GO:0016310	JAK2 JAK3 MET ITK FLT3 KDR CDK9 EGFR PDK1 DYRK1A NTRK1 MTOR NA HSP90AB1 MAPK9 HSP90AA1 PIK3CG MAPK8 PIK3CA RAF1 PPARG PLK2 BRAF PLK1 MAP3K5 DDX3X PTGS2 MAPK10 CCKBR ADORA2A ADAM10 PDE5A DRD2 WEE1 STAT3 HDAC2
5.40E-18	35	1684	6.36645	GO:0006468 protein phosphorylation	http://amigo.geneontology.org/amigo/term/GO:0006468	JAK2 JAK3 ITK CDK9 PDK1 DYRK1A MTOR NA HSP90AB1 MAPK9 HSP90AA1 PIK3CG MAPK8 KDR RAF1 PPARG PLK2 EGFR BRAF PLK1 MAP3K5 NTRK1 DDX3X PTGS2 MET MAPK10 PIK3CA FLT3 ADORA2A

						ADAM10 PDE5A DRD2 WEE1 HDAC2
2.79E-11	34	2795	3.72622	GO:0051246 regulation of protein metabolic process	http://amigo.geneontology.org/amigo/term/GO:0051246	HSP90AB1 HSP90AA1 GZMB PIK3CG KDR RAF1 PPARG CDK9 PLK2 EGFR BRAF PLK1 TYMS MAP3K5 MTOR DDX3X NA NOS2 MAPK9 PTGS2 JAK2 MAPK8 PIK3CA FLT3 ADORA2A PDE5A DRD2 MMP14 DYRK1A HDAC2 NTRK1 STAT3 HDAC1 JAK3
4.48E-11	34	2847	3.65816	GO:0035556 intracellular signal transduction	http://amigo.geneontology.org/amigo/term/GO:0035556	PLK2 NOS2 MAPK9 JAK2 JAK3 PIK3CG MET MAPK8 MAPK10 PIK3CA RAF1 BRAF PDE2A MAP3K5 NTRK1 MTOR KDR PPARG EGFR DRD2 PLK1 DDX3X NA PTGS2 HSP90AA1 HSP90AB1 ITK HDAC1 PDE5A DYRK1A NR3C2 PDK1 FLT3 FPR1
1.78E-12	33	2320	4.35710	GO:0008219 cell death	http://amigo.geneontology.org/amigo/term/GO:0008219	JAK2 JAK3 MAPK8 EGFR MAP3K5 PIK3CA MTOR HSP90AA1 GZMB KDR RAF1 BRAF MAPK9 PTGS2 MMP2 HSP90AB1 PIK3CG NR3C1 HDAC1 ADORA2A PPARG PLK2 SIGMAR1 DRD2 CAPN2 NTRK1 DDX3X NOS2 MET PDK1 PLK1 HDAC2 FLT3
5.16E-10	33	2945	3.43242	GO:0032879 regulation of localization	http://amigo.geneontology.org/amigo/term/GO:0032879	PPARG DRD2 HDAC1 HDAC2 NA HSP90AB1 MAPK8 KDR CDK9 MMP7 EGFR MMP14 BRAF PLK1 PTGS2 HSP90AA1 MMP2 JAK2 JAK3 PIK3CG ADORA2A KCNA5 ADAM10 PLK2 STAT3 NOS2 ABCB1 MET DPP4 MTOR ACHE GZMB RAF1
1.41E-12	32	2127	4.608443	GO:0012501 programmed cell death	http://amigo.geneontology.org/amigo/term/GO:0012501	JAK2 JAK3 MAPK8 EGFR MAP3K5 PIK3CA MTOR HSP90AA1 GZMB KDR RAF1 BRAF MAPK9 PTGS2 MMP2 HSP90AB1 PIK3CG NR3C1 HDAC1 ADORA2A PPARG PLK2 CAPN2 NTRK1 DDX3X NOS2 MET SIGMAR1

						PDK1 PLK1 HDAC2 FLT3
1.61E-12	32	2143	4.574036	GO:0008283 cell population proliferation	http://amigo.geneontology.org/amigo/term/GO:0008283	EGFR STAT3 JAK2 CCKBR KDR RAF1 PPARG DPP4 NTRK1 NOS2 PTGS2 MMP2 PTGS1 JAK3 HDAC1 PIK3CA FLT3 ADORA2A KCNA5 PDE5A DRD2 MMP14 BRAF HDAC2 MAP3K5 ABCB1 ADAM10 PDK1 NAMPT PIK3CG CDK9 S1PR3
1.62E-13	31	1752	5.420001	GO:1901700 response to oxygen-containing compound	http://amigo.geneontology.org/amigo/term/GO:1901700	NOS2 JAK2 JAK3 SLC2A1 DRD2 STAT3 PPARG MAPK8 EGFR PDE2A MAP3K5 MTOR NA PTGS2 HSP90AA1 ACHE MMP2 HSP90AB1 PIK3CG PIK3CA KCNA5 RAF1 SIGMAR1 CAPN2 TYMS HDAC2 NTRK1 MAPK9 MET NR3C1 MMP13
2.64E-13	31	1810	5.246321	GO:0010941 regulation of cell death	http://amigo.geneontology.org/amigo/term/GO:0010941	JAK2 JAK3 MAPK8 EGFR PIK3CA MTOR HSP90AA1 KDR RAF1 BRAF MAP3K5 MAPK9 PTGS2 MMP2 HSP90AB1 PIK3CG NR3C1 HDAC1 ADORA2A PPARG PLK2 DRD2 CAPN2 NTRK1 NOS2 MET SIGMAR1 PLK1 DDX3X HDAC2 FLT3

Figure S17. The original images of Western blot in Figure 3C.

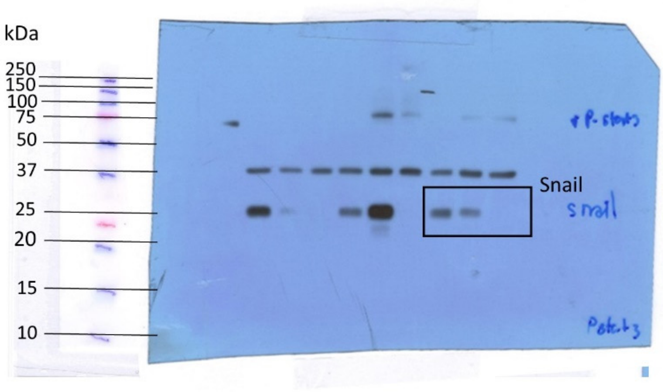

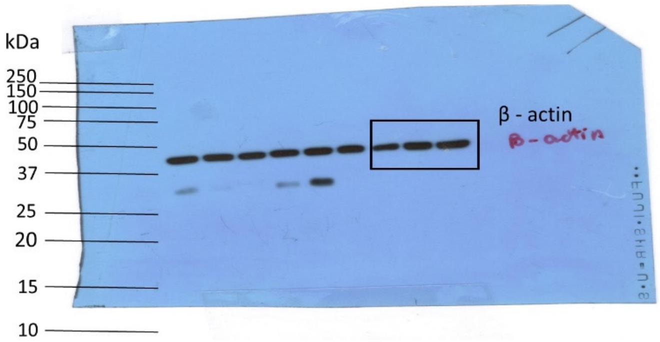

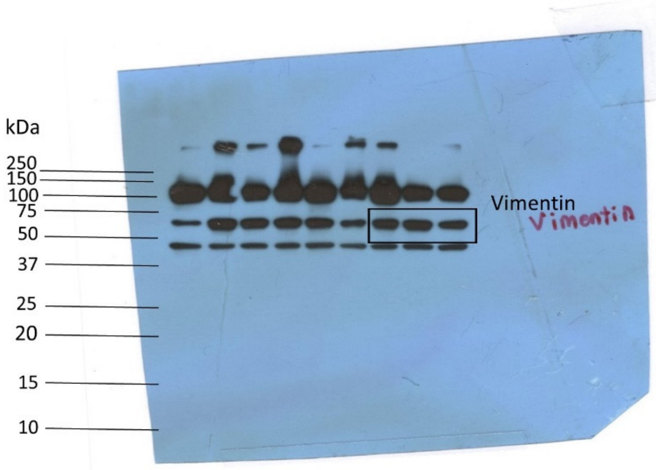

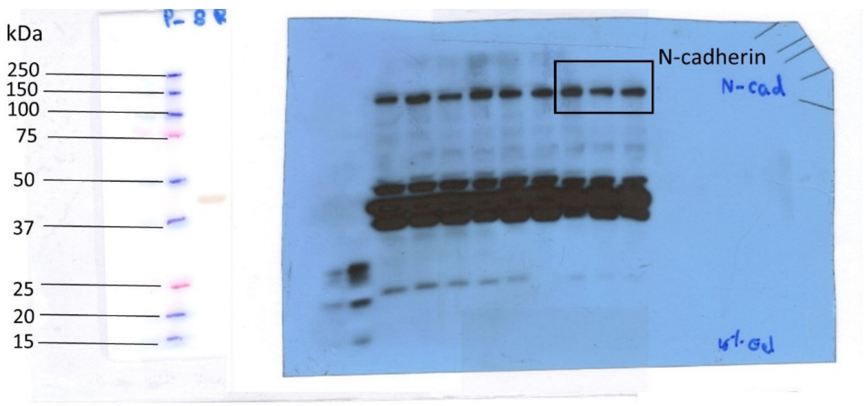
kDa
250
150
100
75
50
37
25
20
15
N-cadherin
N-cad

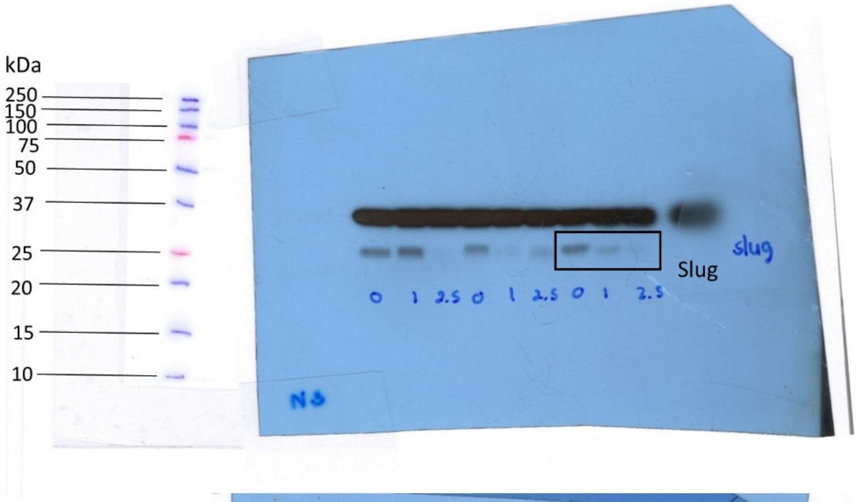
kDa
250
150
100
75
50
37
25
20
15
10
0 1 2.5 0 1 2.5 0 1 2.5
Slug
slug

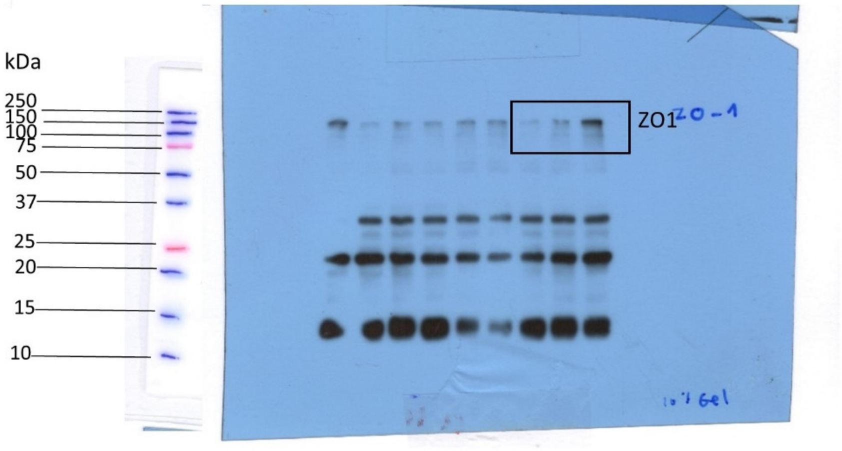
kDa
250
150
100
75
50
37
25
20
15
10
ZO1
ZO-1

Figure S18. The original images of Western blot in Figure 5E.

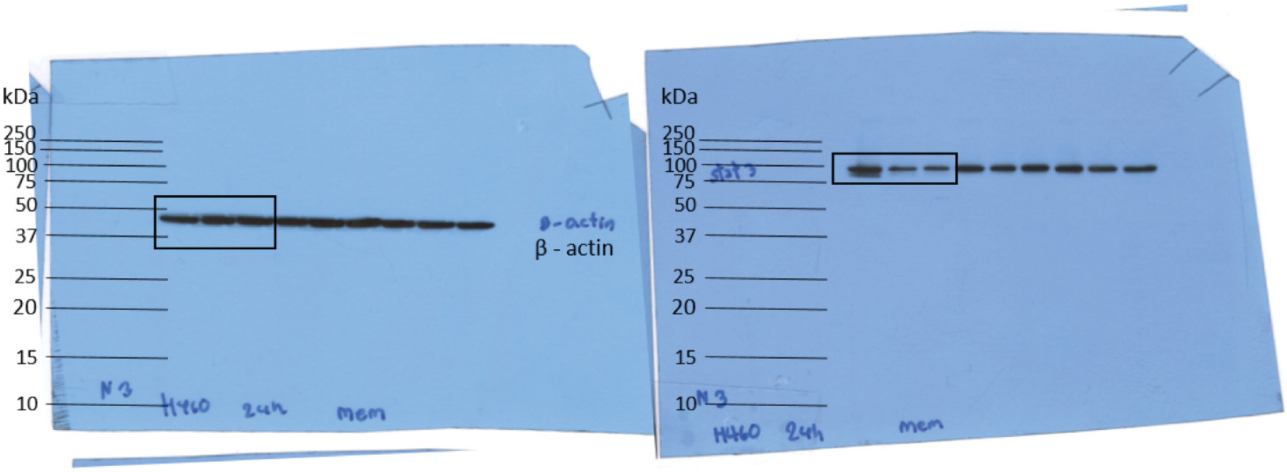

Figure S19. The original images of Western blot in Figure 6B-6D.

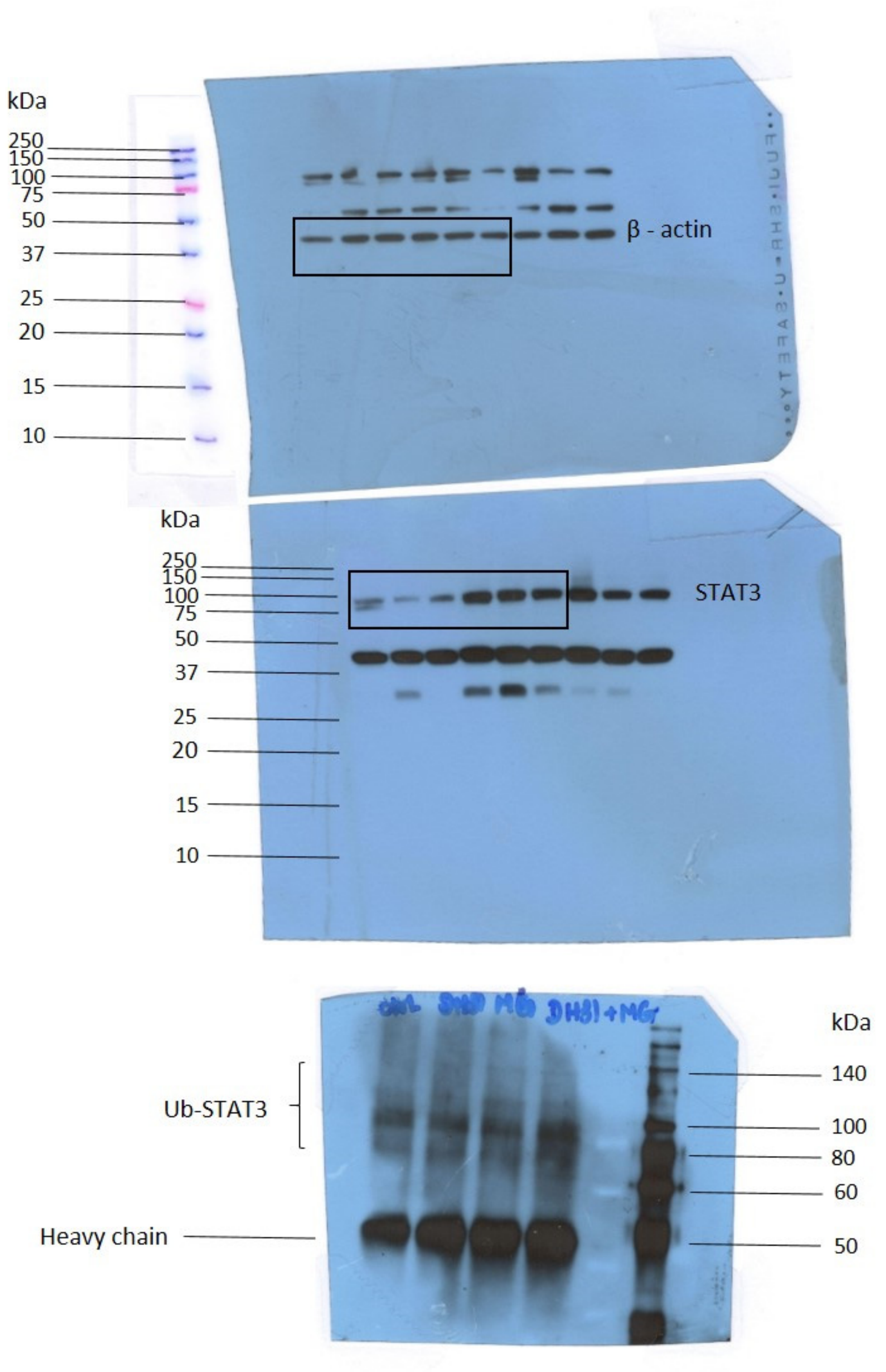

Supplement: Supplementary file 1 [file marinedrugs-22-00370-s001.zip › marinedrugs-3145079-supplementary.pdf]
